# Supplementary material for: Cell surface glycan engineering reveals that matriglycan alone can recapitulate dystroglycan binding and function
Source: Nat Commun. 2022 Jun 24;13:3617. doi: 10.1038/s41467-022-31205-7 (PMC9232514; doi:10.1038/s41467-022-31205-7)
Supplement: Supplementary file 1 — Supplementary Information [file 41467_2022_31205_MOESM1_ESM.pdf]

Supplementary Information: Cell Surface Glycan Engineering Reveals that Matriglycan Alone can Recapitulate Dystroglycan Binding and Function

G.J. Boons *et al.*

Table of Contents

Supplementary Figures……………………………………………………………………………….. S2
Supplementary Methods: Chemical Synthesis………………………………………………….. S7
Supplementary Methods: Enzymatic Synthesis ………………………………………………… S9
Supplementary Figures: NMR Spectra; ESI-MS Data…………………………………………… S13
Supplementary References ………………………………………………………………………. S22

Supplementary Figures

Matriglycans 3:

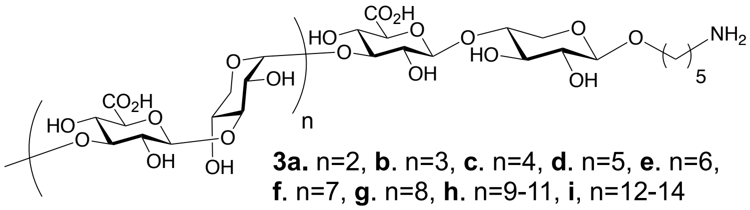

3a. n=2, **b**. n=3, **c**. n=4, **d**. n=5, **e**. n=6, **f**. n=7, **g**. n=8, **h**. n=9-11, **i**, n=12-14

Supplementary Table 1. Observed ESI-MS values for matriglycans **2** and **3a-h** after HPLC purification.

Compound Number	Matriglycan Structure	No. Repeats (n)	Total Residues	ESI-MS m/z calcd	ESI-MS m/z observed
**2**	GlcA-β4-Xyl-β-R	0	2	$[M-H]^-$: 410.1668	410.1659
**3a**	(GlcA-β3-Xyl-α3-)$_2$GlcA-β4-Xyl-β-R	2	6	$[M-H]^-$: 1026.3151	1026.3180
**3b**	(GlcA-β3-Xyl-α3-)$_3$GlcA-β4-Xyl-β-R	3	8	$[M-2H]^{2-}$: 666.6908	666.6937
**3c**	(GlcA-β3-Xyl-α3-)$_4$GlcA-β4-Xyl-β-R	4	10	$[M-2H]^{2-}$: 820.7280	820.7261
**3d**	(GlcA-β3-Xyl-α3-)$_5$GlcA-β4-Xyl-β-R	5	12	$[M-2H]^{2-}$: 974.7652	974.7620
**3e**	(GlcA-β3-Xyl-α3-)$_6$GlcA-β4-Xyl-β-R	6	14	$[M-2H]^{2-}$: 1128.8024	1128.8002
**3f**	(GlcA-β3-Xyl-α3-)$_7$GlcA-β4-Xyl-β-R	7	16	$[M-2H]^{2-}$: 1282.8396	1282.8368
**3g**	(GlcA-β3-Xyl-α3-)$_8$GlcA-β4-Xyl-β-R	8	18	$[M-2H]^{2-}$: 1436.8768<br>$[M-3H]^{3-}$: 957.5819	1436.8768<br>957.5797
**3h**	(GlcA-β3-Xyl-α3-)$_{9-11}$GlcA-β4-Xyl-β-R	9-11	20-24	$[M-3H]^{3-}$: 1060.2734<br>$[M-3H]^{3-}$: 1162.9648<br>$[M-3H]^{3-}$: 1265.6563	1060.2787<br>1162.9620<br>1265.6520

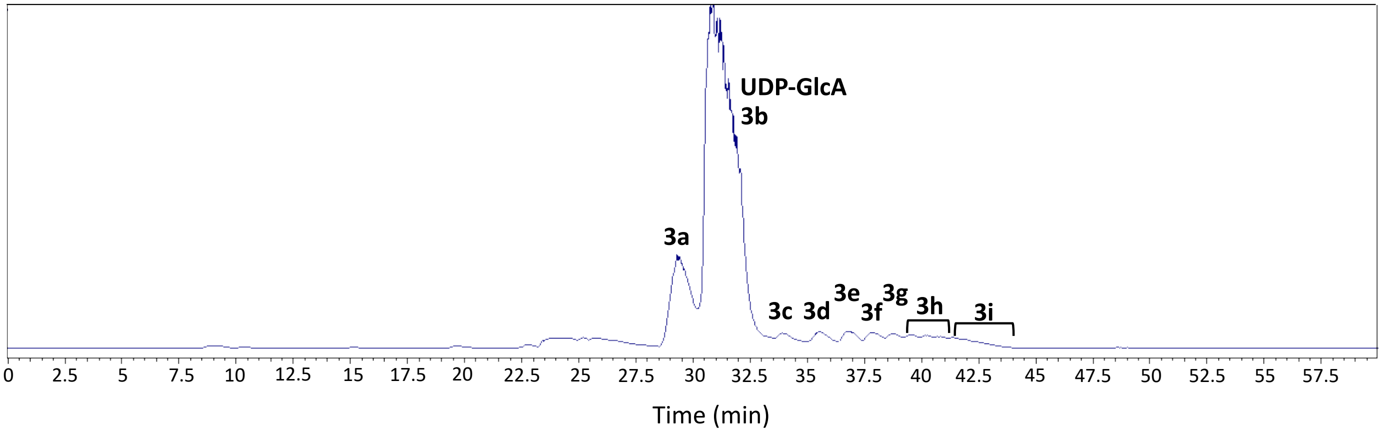

Supplementary Figure 1. HILIC purification matriglycans **3a-j** using a Waters XBridge BEH, Amide column (5 µm, 10 × 250 mm) and the gradient outlined in the LARGE1 extension protocol. Fractions were collected with a volume of approximately 250 µL (20 sec intervals) and products were confirmed by ESI-MS before pooling and lyophilizing.

Matriglycans 8:

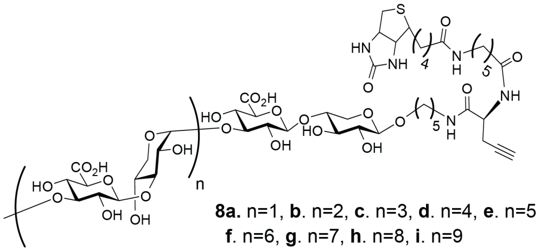

Supplementary Table 2. Observed ESI-MS values for matriglycans **7** and **8a-j** after HPLC purification.

Compound Number	Matriglycan Structure	No. Repeats (n)	Total Residues	ESI-MS m/z calcd	ESI-MS m/z observed
**7**	GlcA-β4-Xyl-β-R	0	2	$[M-H]^-$: 844.3656	844.3635
**8a**	(GlcA-β3-Xyl-α3-)$_1$GlcA-β4-Xyl-β-R	1	4	$[M-H]^-$: 1152.4394<br>$[M-2H]^{2-}$: 575.7158	1152.4332<br>575.7135
**8b**	(GlcA-β3-Xyl-α3-)$_2$GlcA-β4-Xyl-β-R	2	6	$[M-2H]^{2-}$: 729.7530<br>$[M-3H]^{3-}$: 486.1660	729.7535<br>486.1641
**8c**	(GlcA-β3-Xyl-α3-)$_3$GlcA-β4-Xyl-β-R	3	8	$[M-2H]^{2-}$: 883.7902	883.7926
**8d**	(GlcA-β3-Xyl-α3-)$_4$GlcA-β4-Xyl-β-R	4	10	$[M-2H]^{2-}$: 1037.8274<br>$[M-3H]^{3-}$: 691.5490	1037.8240<br>691.5457
**8e**	(GlcA-β3-Xyl-α3-)$_5$GlcA-β4-Xyl-β-R	5	12	$[M-2H]^{2-}$: 1191.8646<br>$[M-3H]^{3-}$: 794.2404	1191.8698<br>794.2440
**8f**	(GlcA-β3-Xyl-α3-)$_6$GlcA-β4-Xyl-β-R	6	14	$[M-3H]^{3-}$: 896.9319<br>$[M-4H]^{4-}$: 672.4470	896.9355<br>672.4491
**8g**	(GlcA-β3-Xyl-α3-)$_7$GlcA-β4-Xyl-β-R	7	16	$[M-3H]^{3-}$: 999.6234<br>$[M-4H]^{4-}$: 749.4656	999.6265<br>749.4630
**8h**	(GlcA-β3-Xyl-α3-)$_8$GlcA-β4-Xyl-β-R	8	18	$[M-3H]^{3-}$: 1102.3148<br>$[M-4H]^{4-}$: 826.4842	1102.3101<br>826.4816
**8i**	(GlcA-β3-Xyl-α3-)$_9$GlcA-β4-Xyl-β-R	9	20	$[M-3H]^{3-}$: 1205.0063	1205.0009

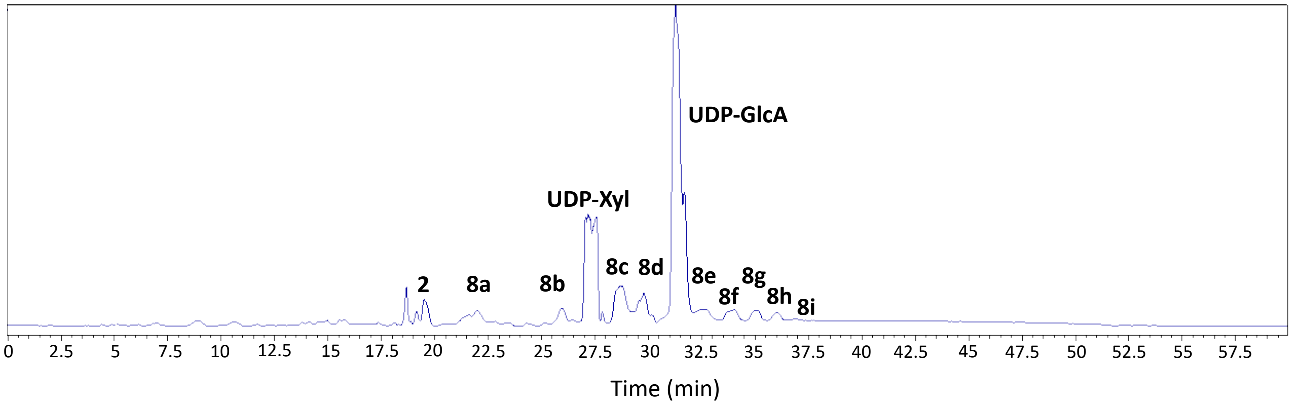

Supplementary Figure 2. HILIC purification matriglycans **8a-j** using a SeQuant ZIC-HILIC Amide column (5 μm, 10 × 250 mm) and the gradient outlined in the LARGE1 extension protocol. Fractions were collected with a volume of approximately 250 μL (20 sec intervals) and products were confirmed by ESI-MS before pooling and lyophilizing.

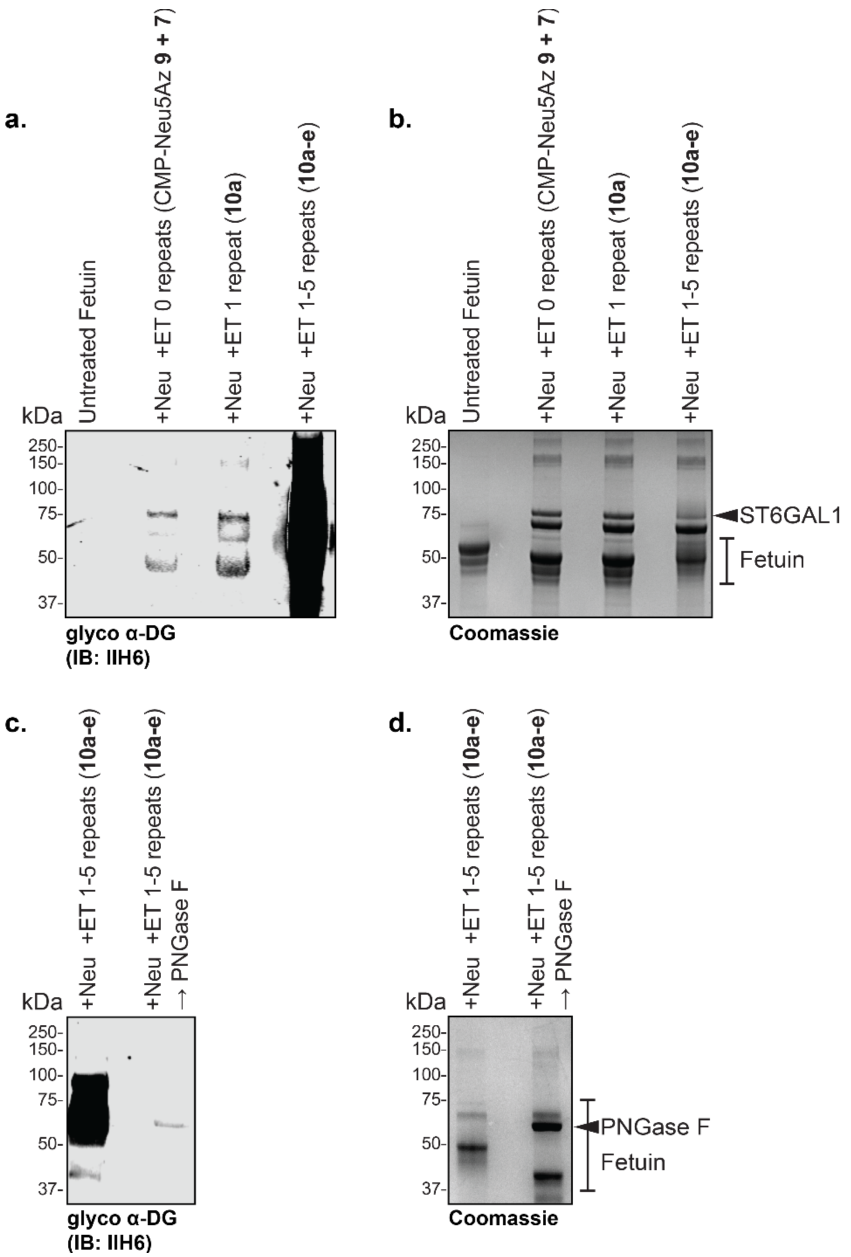

Supplementary Figure 3. Binding of IIH6 antibody to fetuin is matriglycan length-dependent and PNGase F-sensitive (**a**) Western blot analysis matriglycan-engineered fetuin with the anti-glyco-α-DG antibody IIH6. Intensity of IIH6 signal is set to the signal-to-noise threshold for untreated fetuin. IIH6 binding to the Xyl-GlcA primer (0 repeats; CMP-Neu5Az **9** + **7**) is extremely low, slightly increased with 1 repeat (**10a**), and intense binding is observed with a mixture of 1-5 repeats of matriglycan (**10a-e**). (**b**) Coomassie stained SDS-PAGE gel of samples from (a) demonstrating loading of fetuin and ST6GAL1 in each experiment. (**c**) Western blot analysis of fetuin labeled with 1-5 repeats of matriglycan -/+ treatment with PNGaseF demonstrates that the IIH6 signal is dependent on N-linked glycans on fetuin. (**d**) Coomassie stained SDS-PAGE gel of samples from (c) demonstrating the addition of PNGase F and the deglycosylation of fetuin based on the molecular weight shift. *+Neu: +Neuraminidase, +ET: Enzymatic Transfer by ST6GAL1.* Compound identifiers are defined in **Figure 3** and indicated in parentheses; ET with primer disaccharide only labeled as 0 repeats (CMP-Neu5Az **9** + **7**). Experiments were performed in triplicate with similar results each time.

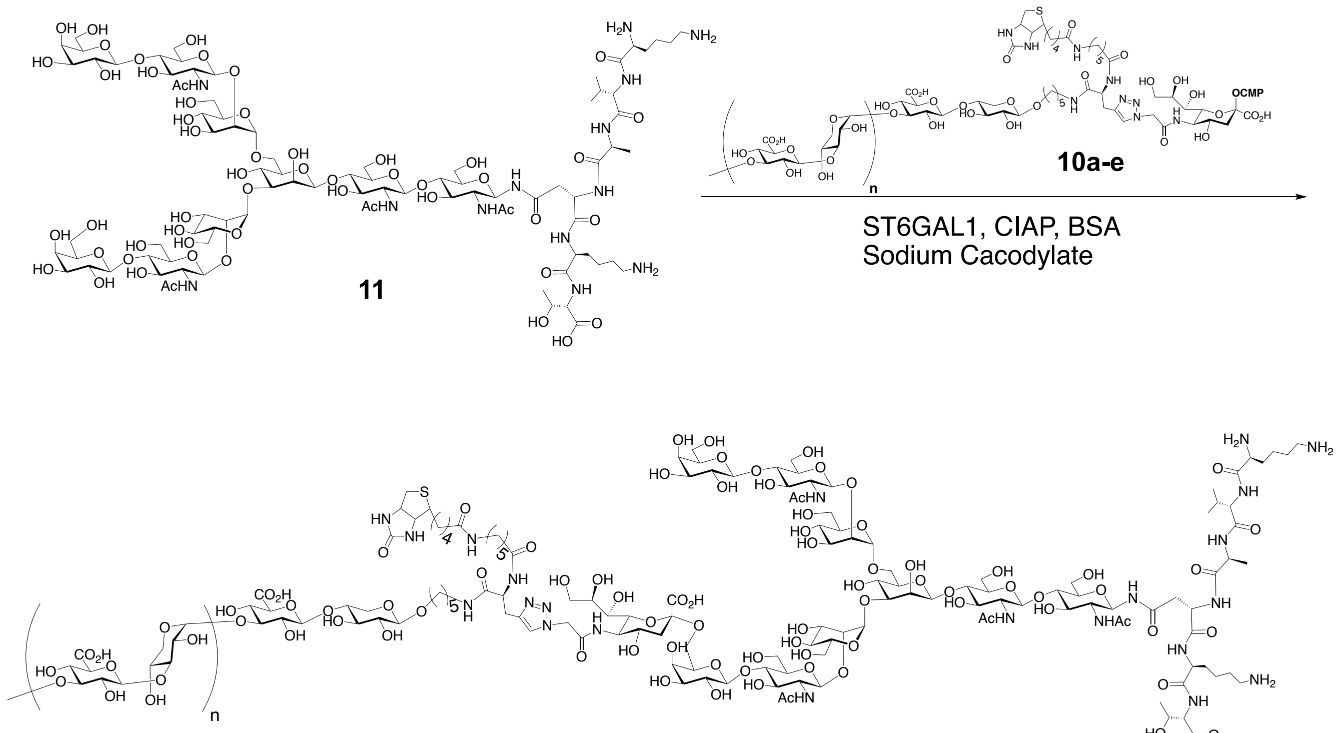

12a. n=1, **b.** n=2, **c**. n=3, **d**. n=4, **e.** n=5

Supplementary Figure 4. Enzymatic transfer of matriglycan-modified CMP-Neu5Ac. Compounds **10a-e** are transferred onto N-glycan substrate **11** using ST6GAL1.

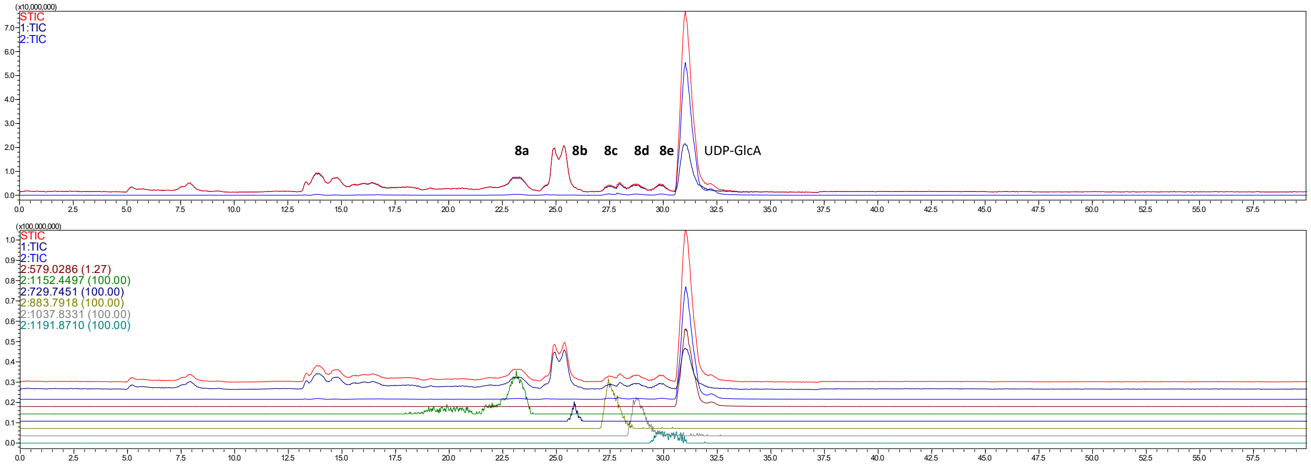

Supplementary Figure 5. HILIC analysis of matriglycans **8a-e** purified by P2-BioGel using a SeQuant ZIC-HILIC Amide column (3.5 μm, 2.1 × 150 mm). The following gradient was used for the analysis: Mobile phase A was ammonium formate in water (10 mM, adjusted to pH 3.4 with formic acid); Mobile phase B was 100% acetonitrile. 1) Gradient of 85% mobile phase B from 0 – 5 min; 2) gradient of 85% to 30% mobile phase B from 5 - 40 min; 3) 30% mobile phase B from 40 - 50 min; 4) gradient of 30% to 85% mobile phase B from 50 - 55 min. 5) gradient of 85% mobile phase B from 55 - 60 min.

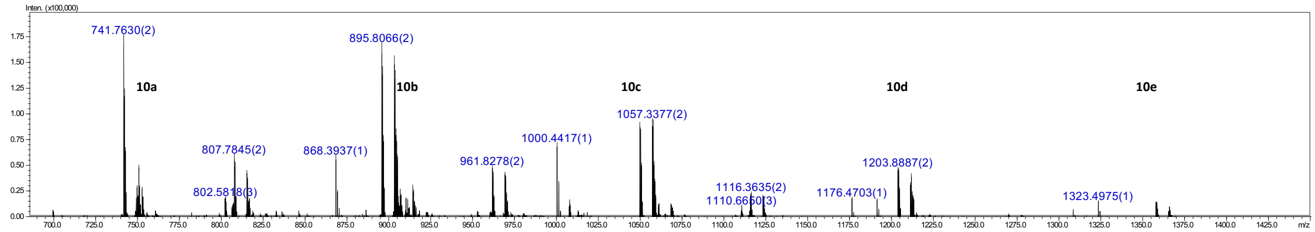

Supplementary Figure 6. ESI-MS analysis of matriglycan modified CMP-Neu5Ac's **10a-e** mixture.

Supplementary Table 3 Observed ESI-MS values for matriglycan modified N-glycans **12a-e** after enzymatic transfer to **11** with ST6GAL1.

Compound Number	Matriglycan Structure	No. Repeats (n)	Total Residues	MSI-MS m/z calcd	ESI-MS m/z observed
**11**	--	--	--	$[M-2H]^{2-}$:1139.9818	1139.9856
**12a**	(GlcA-β3-Xyl-α3-)1GlcA-β4- Xyl-β-R	0	2	$[M-3H]^{3-}$:1152.4765	1152.4741
**12b**	(GlcA-β3-Xyl-α3-)2GlcA-β4- Xyl-β-R	1	4	$[M-3H]^{3-}$:1255.1679	1255.1659
**12c**	(GlcA-β3-Xyl-α3-)3GlcA-β4- Xyl-β-R	2	6	$[M-3H]^{3-}$:1357.8594	1357.8544
**12d**	(GlcA-β3-Xyl-α3-)4GlcA-β4- Xyl-β-R	3	8	$[M-3H]^{3-}$:1460.5508	1460.5360
**12e**	(GlcA-β3-Xyl-α3-)5GlcA-β4- Xyl-β-R	4	10	$[M-3H]^{3-}$:1563.2422	1563.2573

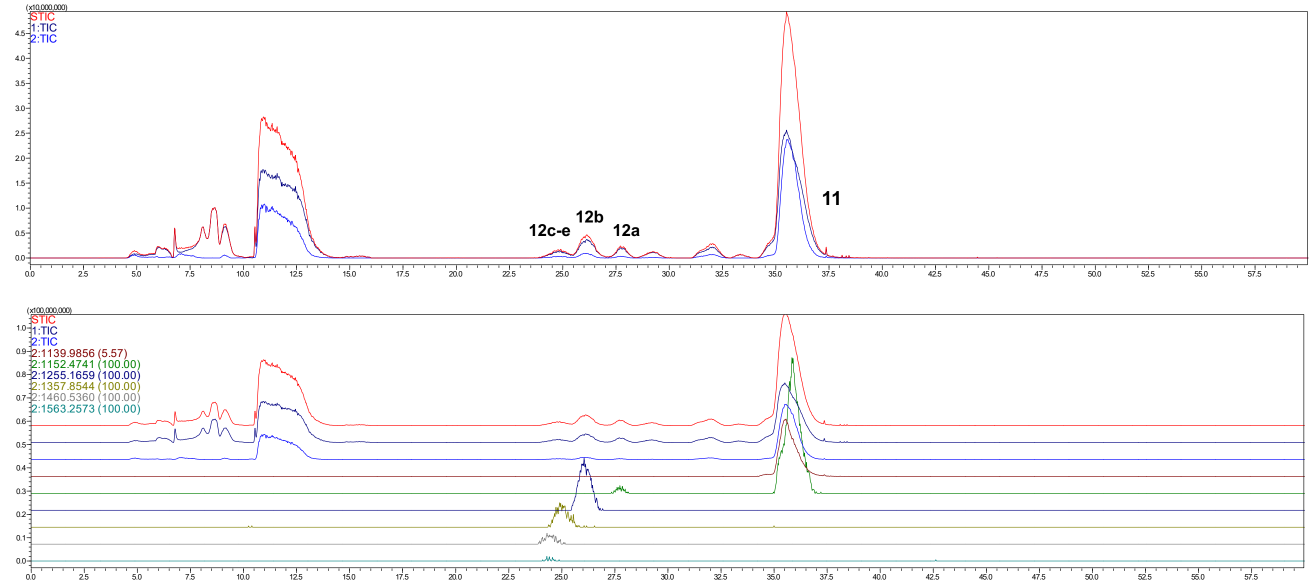

Supplementary Figure 7. HILIC analysis of matriglycan modified N-glycans **12a-e** using a SeQuant ZIC-HILIC Amide column (3.5 μm, 2.1 ╳ 150 mm) and the gradient outlined in Supplementary Fig 5.

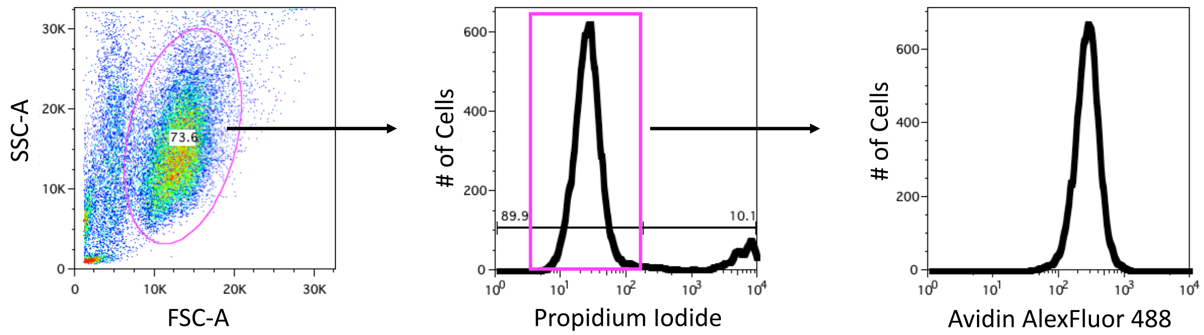

Supplementary Figure 8. Representative gating strategy for analysis of biotinylation of HAP1-*DAG1*⁻ cells after engineering with CMP-Neu5Ac derivative **10e** (100 μM) and ST6GAL1 in the presence of *C. perfringens* neuraminidase.

Supplementary Methods:

Chemical Synthesis

General methods and materials

^{1}H and ^{13}C (data from HSQC) NMR spectra were recorded on a Varian INOVA 300 MHz (^{13}C, 75 MHz), Varian INOVA 500 MHz, a Varian INOVA 600 MHz or an Agilent 900 MHz DD2 spectrometer with a triple resonance (HCN) cryogenically cooled probe spectrometer. Chemical shifts are reported in parts per million (ppm) relative to residual solvent signals used as the internal standard. NMR data is presented as follows: chemical shift, multiplicity (s = singlet, d = doublet, t = triplet, dd = doublet of doublet, m = multiplet and/or multiple resonances), integration, coupling constant in Hertz (Hz). All NMR signals were assigned on the basis of ^{1}H NMR, COSY, zTOCSY, gHSQCAD and gHMBCAD experiments. Mass spectra were recorded on a Shimadzu LCMS-IT-TOF mass spectrometer or an Orbitrap Fusion Tribrid mass spectrometer (Thermo Fisher Scientific). Reagents were purchased from Sigma-Aldrich (unless otherwise noted) and used without further purification. CH_2Cl_2 was freshly distilled from calcium hydride under nitrogen prior to use. Molecular sieves (4Å) were flame activated under vacuum prior to use. All moisture sensitive reactions were carried out under an argon atmosphere. HILIC-HPLC purification of compounds was performed on a Shimadzu 20AD UFLC LCMS-IT-TOF with a Waters XBridge BEH, Amide column, 5 µm, 10 x 250 mm or a SeQuant® ZIC®-HILIC column, 5 µm, 10 x 250 mm. HPLC grade acetonitrile and water were purchased from Fischer. β-Galactoside α-2,6-sialyltransferase 1 (ST6GAL1) was generously provided by Dr. Kelley W. Moremen (Complex Carbohydrate Research Center, University of Georgia, Athens, GA, USA). Calf intestinal alkaline phosphatase (CIAP) was purchased from sigma. *Clostridium perfringens* (*C. perfringens*) neuraminidase was purchased from New England BioLabs. UDP-Glucuronic Acid was purchased from Sigma. UDP-Xylose was purchased from Carbosource (University of Georgia).

Preparation of Xylose-Derivative 1

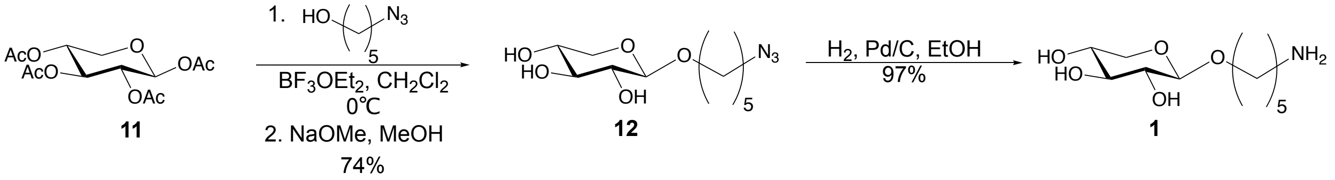

5'-Azidopentyl-β-D-xylopyranoside (12)

1,2,3,4-tetra-*O*-acetyl-β-D-xylopyranoside **11** (1.5 g, 4.7 mmol) and 5-azidopentanol (913 mg, 7.1 mmol) were dissolved in anhydrous CH_2Cl_2 (20 mL) with 4Å molecular sieves and stirred under argon for 30 mins. The mixture was cooled to 0°C and boron trifluoride diethyletherate (1.74 mL, 14.1 mmol) was added dropwise over 15 min and the reaction was stirred while slowly warming to room temperature overnight. The reaction mixture was then diluted with CH_2Cl_2, filtered through Celite, washed with saturated $NaHCO_3$, brine, then dried with $MgSO_4$, filtered, and then concentrated *in vacuo*. The crude product was dissolved in a solution of sodium methoxide in methanol (5 mL) and stirred for one hour at room temperature. The solution was then neutralized with Amberlite® IR-120 (H^+) ion-exchange resin, filtered and concentrated. Purification by silica gel column chromatography (95:5 CH_2Cl_2/MeOH) afforded **12** (905 mg, 74%) as a white solid. ^1H NMR (300 MHz, D_2O) δ 4.41 (d, J = 7.9 Hz, 1H, H1), 3.96 (dd, J = 11.6, 5.4 Hz, 1H, $H5_{eq}$), 3.91 – 3.85 (m, 1H, $OC\underline{\mathit{H}_2}CH_2$), 3.74 – 3.60 (m, 2H, $H5_{ax}$, $OC\underline{\mathit{H}_2}CH_2$), 3.44 (t, J = 9.2 Hz, 1H, H3), 3.39 – 3.30 (m, 3H, H4, $CH_2C\underline{\mathit{H}_2}N_3$), 3.25 (dd, J = 9.3, 7.9 Hz, 1H, H2), 1.72 – 1.60 (m, 4H, $OCH_2C\underline{\mathit{H}_2}$, $C\underline{\mathit{H}_2}CH_2N_3$).), 1.51 – 1.40 (m, 2H, $OCH_2CH_2C\underline{\mathit{H}_2}$). ^{13}C NMR (75 MHz, D_2O) δ 102.9, 75.7, 72.9, 70.3, 69.1, 65.0, 51.0, 28.3, 27.6, 22.3. ESI-MS m/z calcd for $C_{10}H_{18}N_3NaO_5$, $[M+Na]^+$: 284.1217, found 284.1206.

5'-Aminopentyl-β-D-xylopyranoside (1)

Compound **12** (100 mg, 0.38 mmol) was dissolved in a 4:1 ethanol/water mixture (2 mL) and to this palladium on carbon (2 mg, 20% *wt*) was added. The reaction was stirred vigorously under an atmosphere of hydrogen (1 atm) and monitored by ESI-MS until no starting material could be detected. Once complete, the reaction was filtered through a Whatman ® syringe filter (0.2 micron) to remove the catalyst and the filtrate was lyophilized to yield **1** (86 mg, 97%) as a white solid. ^1H NMR (300 MHz, D_2O) δ 4.41 (d, J = 7.9 Hz, 1H, H1), 3.95 (dd, J = 11.5, 5.4 Hz, 1H, $H5_{eq}$), 3.91 – 3.82 (m, 1H, $OC\underline{\mathit{H}_2}CH_2$), 3.73 – 3.56 (m, 2H, $H5_{ax}$, $OC\underline{\mathit{H}_2}CH_2$), 3.44 (t, J = 9.2 Hz, 1H, H3), 3.37 – 3.20 (m, 2H, H4, H2), 2.65 (t, J = 6.9 Hz, 2H, $CH_2C\underline{\mathit{H}_2}NH_2$), 1.64 (m, 2H, $OCH_2C\underline{\mathit{H}_2}$), 1.56 – 1.31 (m, 4H, $C\underline{\mathit{H}_2}CH_2NH_2$, $OCH_2CH_2C\underline{\mathit{H}_2}$). ^{13}C NMR (75 MHz, D_2O) δ 102.9, 75.7, 73.0, 70.5, 69.1, 65.0, 40.2, 30.8, 28.5, 22.4. ESI-MS m/z calcd for $C_{10}H_{20}NO_5$, $[M-H]^-$: 234.2725, found 234.2717.

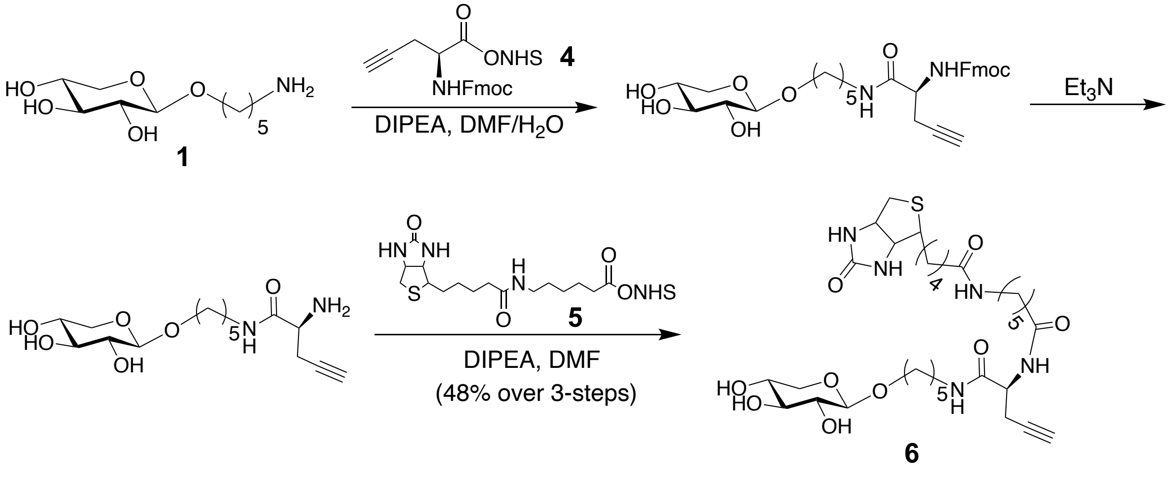

Xylose Derivative 6

Compound **1** (50 mg, 0.213 mmol), NHS-activated Fmoc-propargyl glycine **4**[1] (110.3 mg, 0.255 mmol) and DIPEA (74 µL, 0.426 mmol) were dissolved in DMF (2 mL). The reaction mixture was stirred at room temperature until **1** could no longer be detected by ESI-MS. Next, 100 uL triethylamine was added and the mixture was stirred for 30 minutes. After removing solvent under reduced pressure, the crude product was dissolved in DMF (1 mL) followed by the addition of EZ-Link NHS-LC-Biotin **5** (106 mg,

0.234 mmol) and DIPEA (74 µL, 0.426 mmol). After completion of the reaction was indicated by ESI-MS, the mixture was concentrated under reduced pressure. Purification by silica gel column chromatography by a gradient (9:1:0.5 to 7:2:1 v/v/v, EtOAc/MeOH/H_2O) afforded **6** (68 mg, 48%) as a white solid. ^{1}H NMR (500 MHz, D_2O) δ 4.63 (dd, *J* = 7.9, 4.9 Hz, 1H, C*H*CH_2-Biotin), 4.48 – 4.37 (m, 3H, H1-Xyl, C*H*CH-Biotin, C*H*-propargyl-glycine), 3.95 (dd, *J* = 11.2, 5.4 Hz, 1H, H5$_{eq}$), 3.86 (m, 1H, OC*H*$_2$$CH_2$), 3.73 – 3.57 (m, 2H, H5$_{ax}$, OC*H*$_2$$CH_2$), 3.44 (t, *J* = 9.1 Hz, 1H, H3), 3.35 (m, 2H, H4-Xyl, CHC*H*S-Biotin,), 3.30 – 3.14 (m, 5H, H2-Xyl, C*H*$_2$NH, C*H*$_2$NH), 3.02 (dd, *J* = 13.3, 5.0 Hz, 1H, CHC*H*$_2$-Biotin), 2.80 (d, *J* = 12.8 Hz, 1H, CHC*H*$_2$-Biotin), 2.70 (dd, *J* = 6.4, 2.0 Hz, 2H, C*H*$_2$CCH), 2.51 – 2.46 (m, 1H, CH_2CC*H*), 2.34 (t, *J* = 7.4 Hz, 2H, C*H*$_2$CO), 2.27 (t, *J* = 7.1 Hz, 2H, C*H*$_2$CO), 1.85 – 1.48 (m, 12H, C*H*$_2$-linker), 1.48 – 1.24 (m, 6H, C*H*$_2$-linker). ^{13}C NMR (126 MHz, D_2O) δ 176.89, 176.53, 171.85, 102.88, 79.27, 75.73, 72.96, 72.15, 70.38, 69.13, 65.04, 62.02, 60.18, 55.32, 52.52, 39.63, 39.13, 39.02, 35.45, 35.14, 28.32, 27.95, 27.79, 27.61, 25.48, 25.13, 24.79, 22.32, 21.09. ESI-MS m/z calcd for $C_{31}H_{50}N_5O_9S$, $[M-H]^-$: 668.3335, found 668.3321.

Enzymatic Synthesis

General procedure for the installation of β1,4-GlcA using B4GAT1

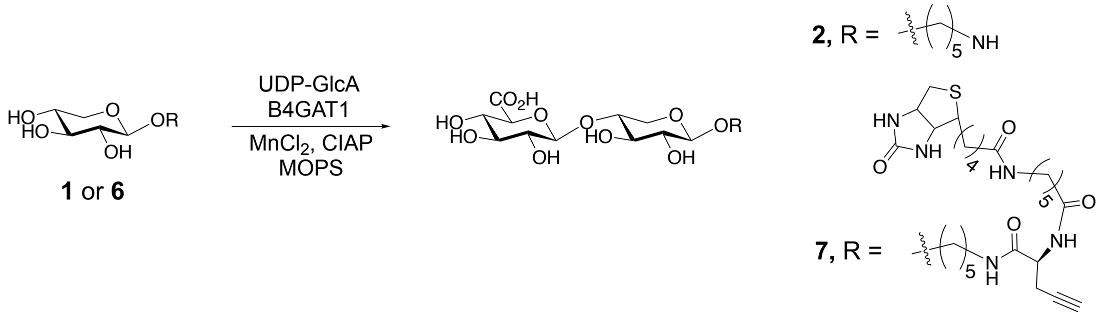

Xylose acceptor (10.6 µmol) and UDP-GlcA (15.9 µmmol) were dissolved at a final xylose-derivative concentration of 10 mM in a MOPS buffered solution (100 mM, pH 7.0) containing $MnCl_2$ (10 mM). CIAP (1% total volume) and B4GAT1 (43 µg/µmol acceptor) were added, and the reaction mixture was incubated overnight at 37°C with gentle shaking. Reaction progress was monitored by ESI-MS and if starting material remained after 18 h another portion of B4GAT1 was added until no starting material could be detected. The reaction mixture was centrifuged using a Nanosep® Omega ultrafiltration device (10 kDa MWCO) to remove enzymes and the filtrate was lyophilized. The residue was purified by HPLC using a SeQuant ZIC-HILIC Amide column (5 µm, 10 × 250 mm) with 1% of the flow diverted to the ESI-MS detector. Mobile phase A was ammonium formate in water (10 mM, adjusted to pH 4.5 with formic acid); Mobile phase B was a mixture of acetonitrile (90%) with ammonium formate in water (10%, 10 mM, pH = 4.5 with formic acid). The following gradient was used to provide the desired product: 1) Gradient of 90% to 60% mobile phase A from 0 - 35 min; 2) gradient of 60% to 30% mobile phase A from 35 - 40 min; 3) 30% mobile phase A from 35 - 55 min; 4) gradient of 30% to 90% mobile phase A from 55 - 60 min.

Disaccharide 2

Xylose acceptor **1** (2.5 mg, 10.6 µmol) was used to prepare disaccharide **2**. Following HPLC purification, fractions containing product were pooled and lyophilized to yield **2** (3.9 mg, 95%) as a white solid.
^{1}H NMR (500 MHz, D_2O) δ 4.55 (d, *J* = 7.9 Hz, 1H, H1-GlcA), 4.44 (d, *J* = 7.9 Hz, 1H, H1-Xyl), 4.10 (dd, *J* = 11.8, 5.4 Hz, 1H, H5$_{eq}$-Xyl), 3.90 (m, 1H, OC*H*$_2$$CH_2$), 3.84 (td, *J* = 9.7, 5.3 Hz, 1H, H4-Xyl), 3.77 – 3.73 (m, 1H, H4-GlcA), 3.70 (m, 1H, OC*H*$_2$$CH_2$), 3.59 (t, *J* = 9.2 Hz, 1H, H3-Xyl), 3.55 – 3.48 (m, 2H, H3-GlcA, H5-GlcA), 3.40 (dd, *J* = 11.8, 10.4 Hz, 1H, H5$_{ax}$-Xyl), 3.36 – 3.27 (m, 2H, H2-GlcA, H2-Xyl), 3.04 – 2.99 (t, *J* = 7.5 Hz, 2H, CH_2C*H*$_2$$NH_2$), 1.69 (m, 4H, C*H*$_2$-linker), 1.47 (m, 2H, C*H*$_2$-linker). ^{13}C NMR (126 MHz, D_2O) δ 102.8, 101.0, 76.6, 75.8, 75.5, 74.0, 73.0, 71.9, 70.1, 62.9, 39.4, 28.3, 26.4, 22.2. ESI-MS m/z calcd for $C_{16}H_{28}NO_{11}$, $[M-H]^-$: 410.1668, found 410.1659.

Disaccharide 7

Xylose acceptor **6** (2.5 mg, 3.7 µmol) was used to prepare disaccharide **7.** Following HPLC purification, fractions containing product were pooled and lyophilized to yield **7** (2.7 mg, 87%) as a white solid.

^{1}H NMR (500 MHz, D_2O) δ 4.63 (dd, *J* = 7.8, 5.0 Hz, 1H, C*H*CH_2-Biotin), 4.55 (d, *J* = 7.9 Hz, 1H, H1-GlcA), 4.43 (m, 3H, H1-Xyl, C*H*CH-Biotin, C*H*-propargyl-glycine), 4.09 (dd, *J* = 11.8, 5.4 Hz, 1H, $H5_{eq}$-Xyl), 3.90 – 3.80 (m, 2H, H4-Xyl, O$C\underline{H}_2CH_2$), 3.75 (m, 1H, H4-GlcA), 3.71 – 3.63 (m, 1H, O$C\underline{H}_2CH_2$), 3.59 (t, *J* = 9.1 Hz, 1H, H3-Xyl), 3.56 – 3.49 (m, 2H, H3-GlcA, H5-GlcA), 3.41 (dd, *J* = 11.2, 10.4 Hz, 1H, $H5_{ax}$-Xyl), 3.39 – 3.20 (m, 7H, H2-Xyl, H2-GlcA, CHC*H*S-Biotin, $C\underline{H}_2$NH, $C\underline{H}_2$NH), 3.02 (dd, *J* = 13.1, 5.0 Hz, 1H, CH$C\underline{H}_2$-Biotin), 2.80 (d, *J* = 13.1 Hz, 1H, $C\underline{H}_2$CCH), 2.73 – 2.66 (m, 2H, $C\underline{H}_2$CCH), 2.48 (t, *J* = 2.6 Hz, 1H, CH_2CC*H*), 2.34 (t, *J* = 7.1 Hz, 2H, $C\underline{H}_2$CO), 2.27 (t, *J* = 7.2 Hz, 2H, $C\underline{H}_2$CO), 1.76 – 1.52 (m, 12H, $C\underline{H}_2$-linker), 1.39 (m, 6H, $C\underline{H}_2$-linker). ESI-MS m/z calcd for $C_{37}H_{58}N_5O_{15}S$, $[M-H]^-$: 844.3656, found 844.3635.

General procedure for disaccharide extension into matriglycan polysaccharides using LARGE1

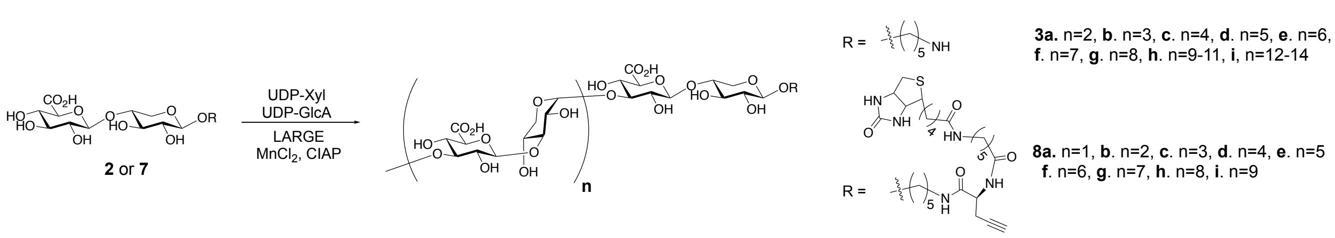

Disaccharide acceptor (2.0 µmol, 1 equivalent) was dissolved at a concentration of 10 mM in a MES buffered solution (100 mM, pH 6.0) containing $MnCl_2$ (10 mM). For shorter matriglycan lengths (n<4), 4 equivalents of UDP-Xyl (8.0 µmol) and 5 equivalents of UDP-GlcA (10.0 µmol) were added to the reaction mixture. For longer matriglycan lengths (n>3), 17 equivalents of UDP-Xyl (34.0 µmol) and 18 equivalents of UDP-GlcA (36.0 µmol) were added to the reaction mixture. UDP-GlcA was used in excess to cap all matriglycans with GlcA. CIAP (1% total volume) and LARGE1 (200 µg/µmol acceptor) were added, and the reaction mixture was incubated overnight at 37°C with gentle shaking. The reaction mixture was centrifuged using a Nanosep® Omega ultrafiltration device (30 kDa MWCO) to remove enzymes and the filtrate was lyophilized.

The residue for reactions yielding matriglycans **8** was purified by HPLC using a SeQuant ZIC-HILIC Amide column (5 µm, 10 × 250 mm). The residue for reactions yielding matriglycans **3** was purified by HPLC using Waters XBridge BEH, Amide column (5 µm, 10 × 250 mm). 1% of the flow diverted to the ESI-MS detector. For all HPLC purifications, mobile phase A was ammonium formate in water (10 mM, adjusted to pH 4.5 with formic acid); Mobile phase B was a mixture of acetonitrile (90%) with ammonium formate in water (10%, 10 mM, pH = 4.5 with formic acid). The following gradient was used for both columns to provide the desired products: 1) Gradient of 90% to 60% mobile phase A from 0 - 35 min; 2) gradient of 60% to 30% mobile phase A from 35 - 40 min; 3) 30% mobile phase A from 35 - 55 min; 4) gradient of 30% to 90% mobile phase A from 55 - 60 min. Fractions were collected with a volume of approximately 250 µL (20 sec intervals) and products were confirmed by ESI-MS before pooling and lyophilizing. HPLC-MS analysis and observed MS values for matriglycans are shown in Supplementary Fig. 1 and Supplementary Table 1 for matriglycans **3** and Supplementary Fig. 2 and Supplementary Table 2 for matriglycans **2**.

General protocol for conjugation of matriglycans to CMP-Neu5Az by CuAAC

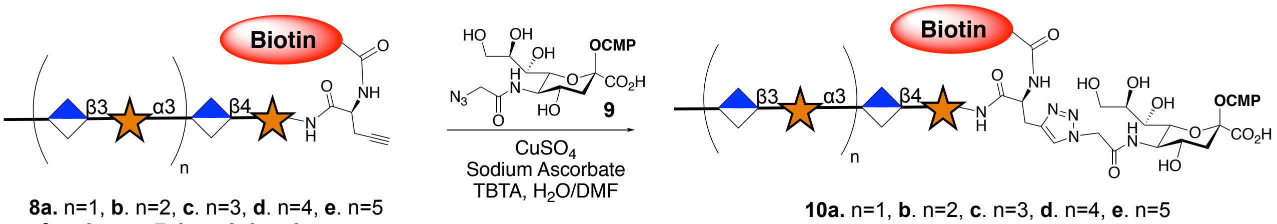

8a. n=1, **b**. n=2, **c**. n=3, **d**. n=4, **e**. n=5
f. n=6, **g**. n=7, **h**. n=8, **i**. n=9

10a. n=1, **b**. n=2, **c**. n=3, **d**. n=4, **e**. n=5
f. n=6, **g**. n=7, **h**. n=8, **i**. n=9

Stock solutions of 0.1 M $CuSO_4$, 0.2 M sodium L-ascorbate and 0.1 M TBTA in 0.1 M NH_4HCO_3 were freshly made before each CuAAC reaction. 2 equivalents of $CuSO_4$ per GlcA-carboxylate residue were used for each reaction. Sodium ascorbate and TBTA were adjusted to $CuSO_4$ quantities at a ratio of 1.5:1 for sodium ascorbate/$CuSO_4$ and 0.5:1 for TBTA/$CuSO_4$. $CuSO_4$, sodium ascorbate and TBTA were pre-mixed by vortexing, and were then added to a solution of alkyne-matriglycans **8a-i** (1 equivalent) and CMP-Neu5Az **9**[2] (3 equivalents) in 100 µL 0.1 M NH_4HCO_3. The resulting mixture was stirred at room temperature for 2 hours to have minimal hydrolysis of the CMP-Neu5Ac-derivative. The mixture was then directly loaded onto a P2-BioGel column kept at 4°C and the product was purified using 0.1 NH_4HCO_3 as eluent, analyzed by ESI-MS and immediately lyophilized and used for glyco-engineering studies.

Transfer of Matriglycan Oligosaccharides to N-linked Glycopeptide 11

Disaccharide acceptor **7** (0.2 µmol) was dissolved at a concentration of 10 mM in a MES buffered solution (100 mM, pH 6.0) containing MnCl2 (10 mM), UDP-Xyl (1.2 µmol) and UDP-GlcA (1.4 µmol). CIAP (1% total volume) and LARGE1 (100 µg/µmol acceptor) were added, and the reaction mixture was incubated overnight at 37 °C with gentle shaking. The reaction mixture was centrifuged using a Nanosep® Omega ultrafiltration device (30 kDa MWCO) to remove the enzymes, and the resulting filtrate was lyophilized. The residue was purified by P-2 Bio-Gel column chromatography using 0.1 NH4HCO3 as eluent. Matriglycan containing fractions were combined and lyophilized to give a mixture of **8a-e** (0.6 mg), which was used without further purification. The mixture of matriglycans **8a-e** after P-2 Bio-Gel purification was analyzed by LC-MS using a SeQuant ZIC-HILIC Amide column (3.5 µm, 2.1 × 150 mm). Mobile phase A was ammonium formate in water (10 mM, adjusted to pH 3.4 with formic acid); Mobile phase B was 100% acetonitrile. The following gradient was used for the analysis: 1) Gradient of 85% mobile phase B from 0 – 5 min; 2) gradient of 85% to 30% mobile phase B from 5 - 40 min; 3) 30% mobile phase B from 40 - 50 min; 4) gradient of 30% to 85% mobile phase B from 50 - 55 min. 5) gradient of 85% mobile phase B from 55 - 60 min. HPLC-MS analysis is shown in Supplementary Fig. 5.

The conjugation of matriglycans (**8a-e** mixture) to CMP-Neu5Az **9** was performed according to the protocol for click-conjugation by CuAAC to yield **10a-e** (0.4 mg). ESI-MS analysis of the **10a-e** mixture is shown in Supplementary Fig. 6.

N-Glycan **11** was used as the acceptor substrate for the matriglycan modified CMP-Neu5Az derivatives **10a-e**.[3] N-glycan **11** (11.4 µg, 0.005 µmol) was dissolved at a concentration of 2 mM in a sodium cacodylate buffered solution (100 mM, pH 6.5). ST6GAL1 (10 µg/mL acceptor, 1 µL), 10 U/mL CIAP (1 µL) and 0.1% BSA (1 µL) were added. After the addition of the **10a-e** mixture (0.05 mg), the reaction mixture was incubated overnight at 37 °C with shaking. Matriglycan-*N*-glycan complexes **12a-e** was analyzed by ESI-LC using a SeQuant ZIC-HILIC Amide column (3.5 µm, 2.1 × 150 mm). 1% of the flow diverted to the ESI-MS detector. For all HPLC separations, mobile phase A was ammonium formate in water (10 mM, adjusted to pH 3.4 with formic acid); Mobile phase B was acetonitrile. The following gradient was used to provide the desired products: 1) Gradient of 65% mobile phase B from 0 – 5 min; 2) gradient of 65% to 30% mobile phase B from 5 - 40 min; 3) 30% mobile phase B from 40 - 50 min; 4)

gradient of 30% to 65% mobile phase B from 50 - 55 min. 5) gradient of 65% mobile phase B from 55 - 60 min. LC-MS analysis and observed MS values for matriglycan-N-glycan **12a-e** complexes are shown in Supplementary Fig. 7 and Supplementary Table 3.

Supplementary Figures: NMR Data

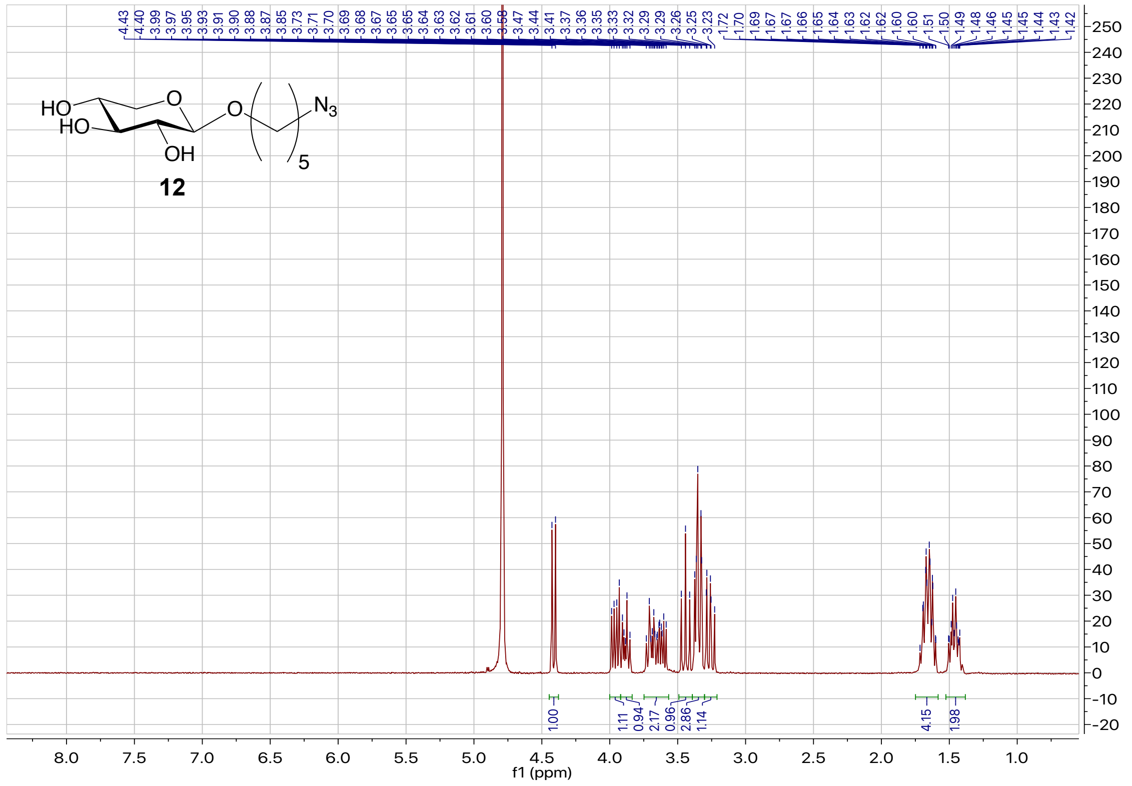

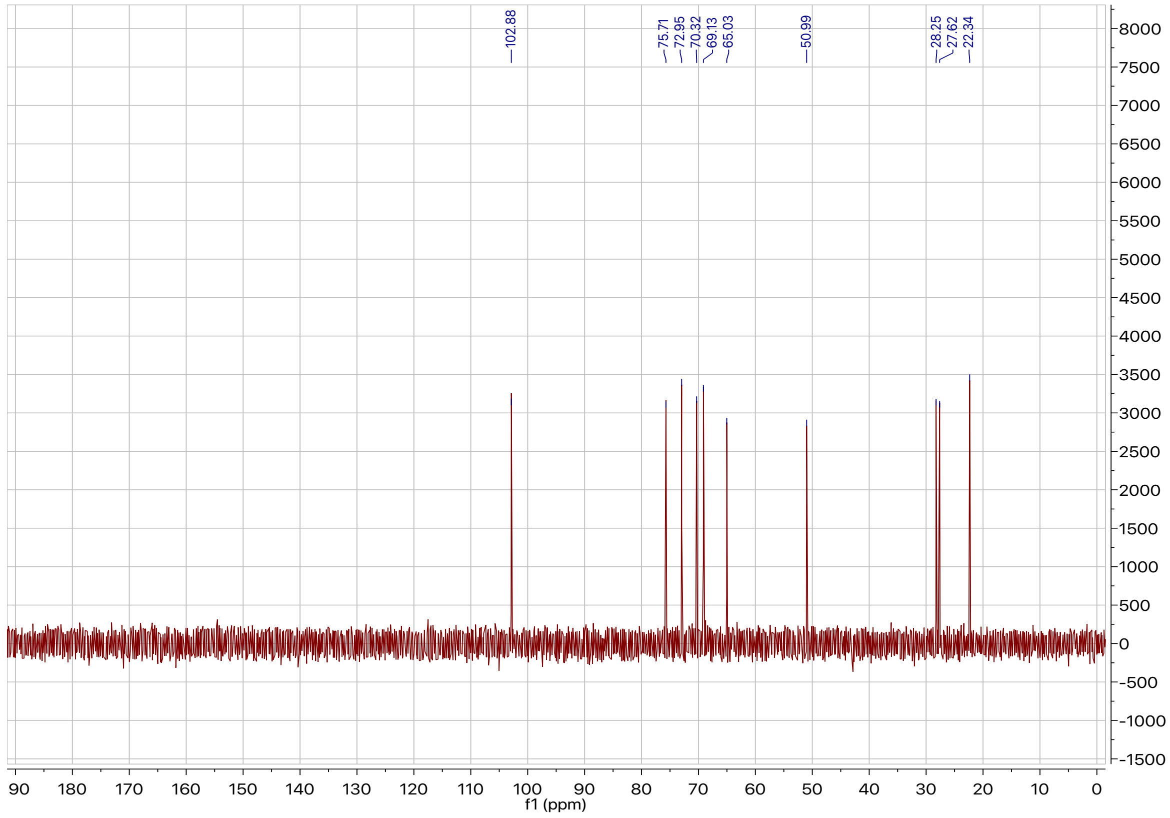

Supplementary Figure 9. ^{1}H (300 MHz, D_2O) and ^{13}C (75 MHz, D_2O) NMR spectra for **12**.

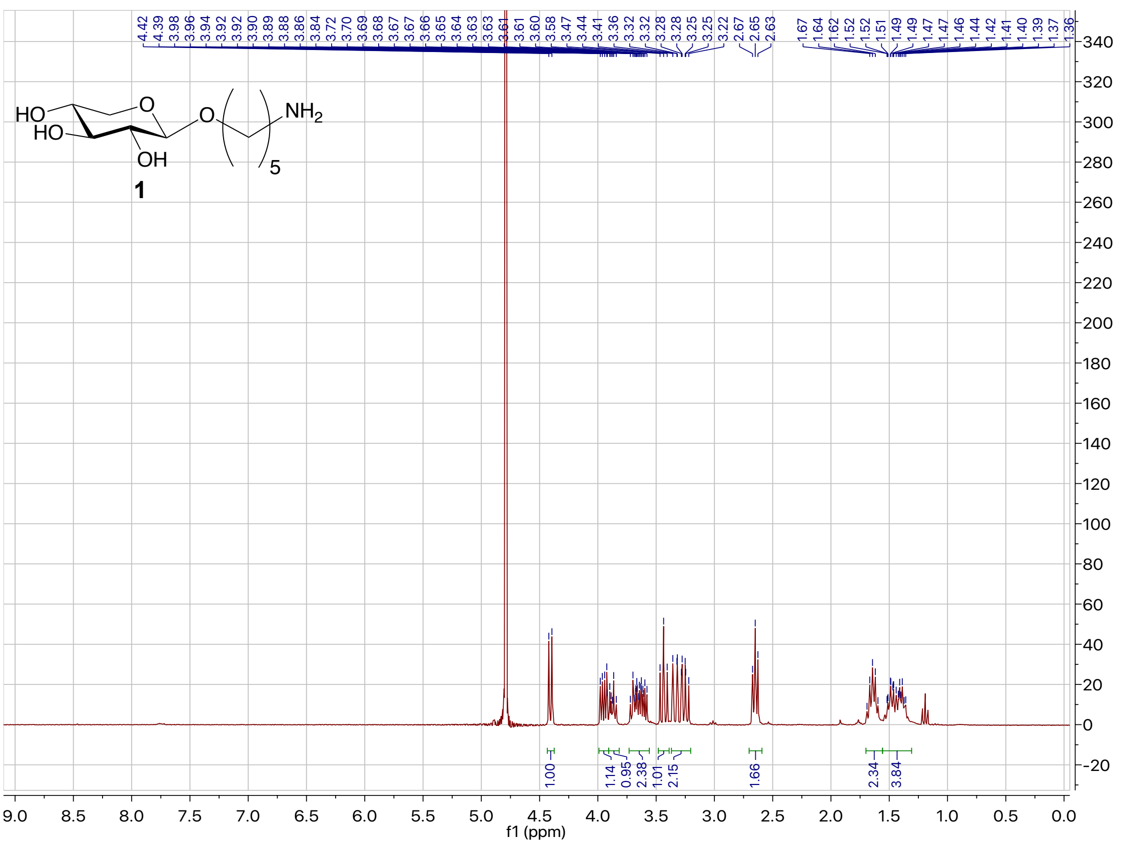

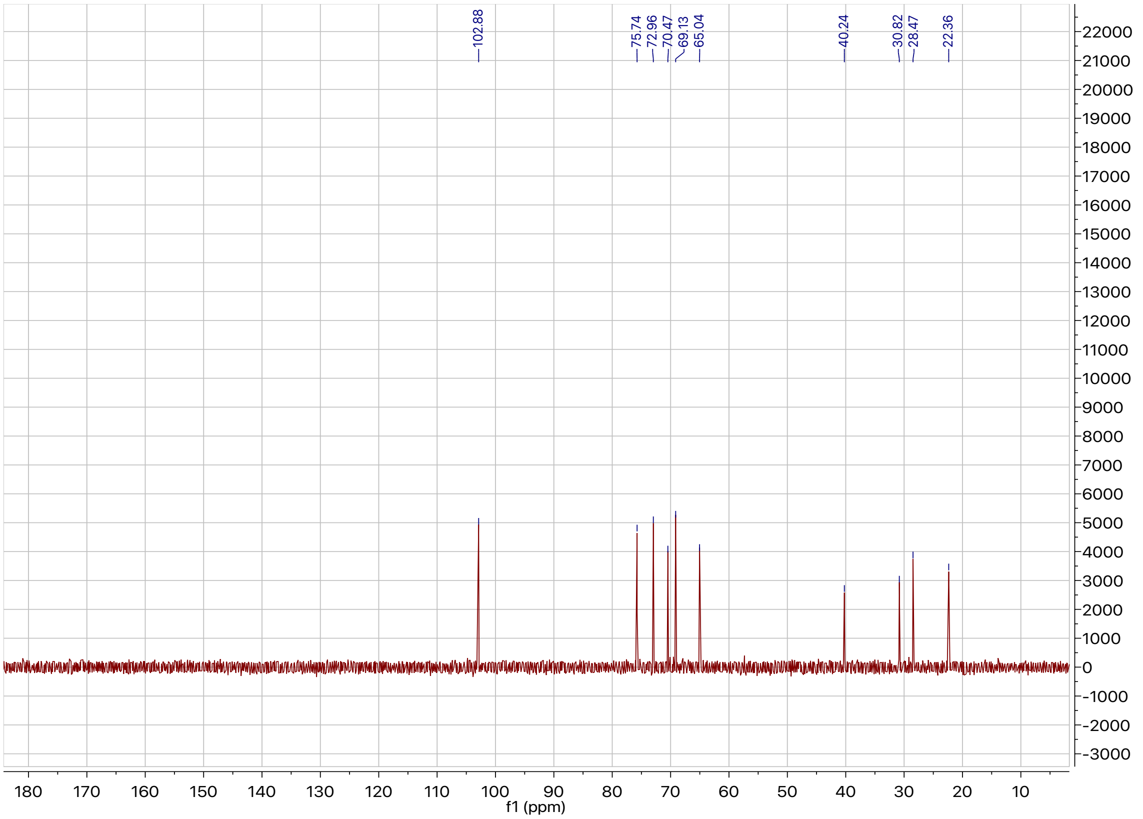

Supplementary Figure 10. ^{1}H (300 MHz, D_2O) and ^{13}C (75 MHz, D_2O) NMR spectra for **1**.

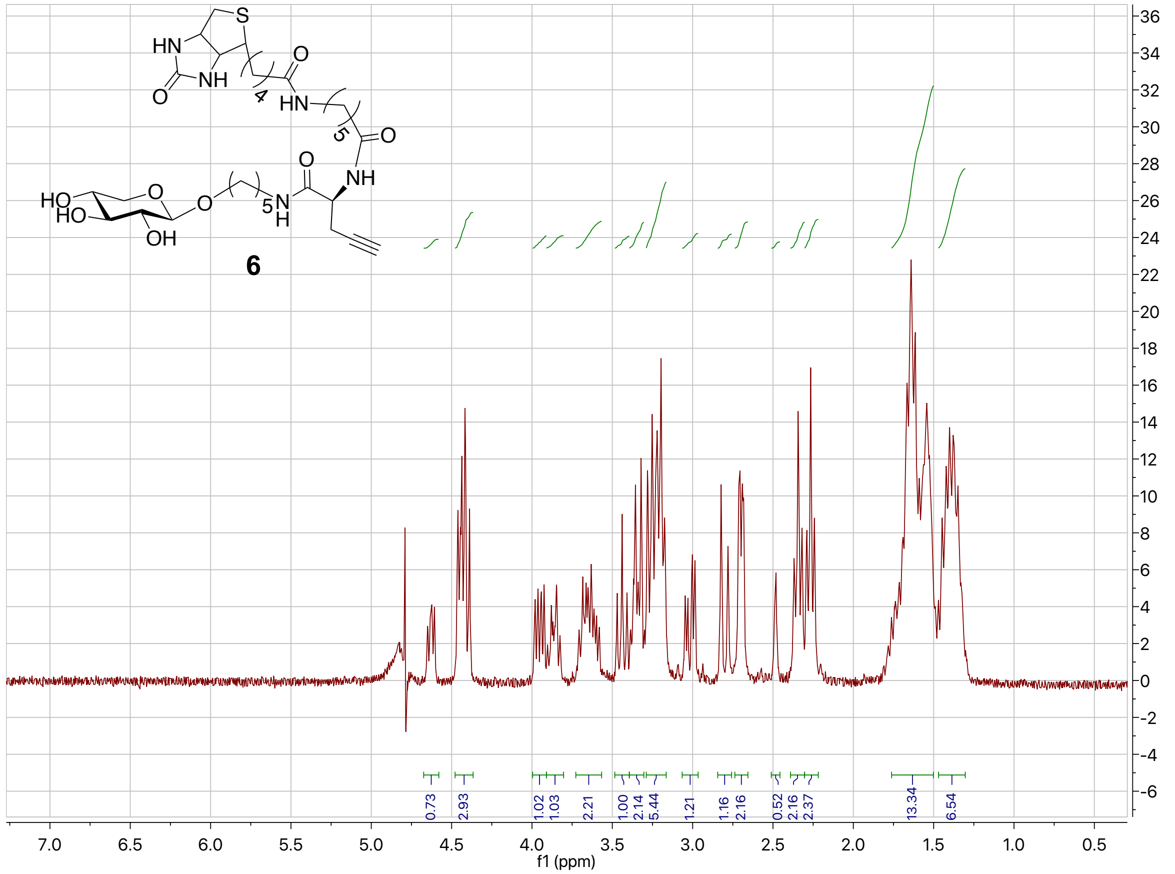

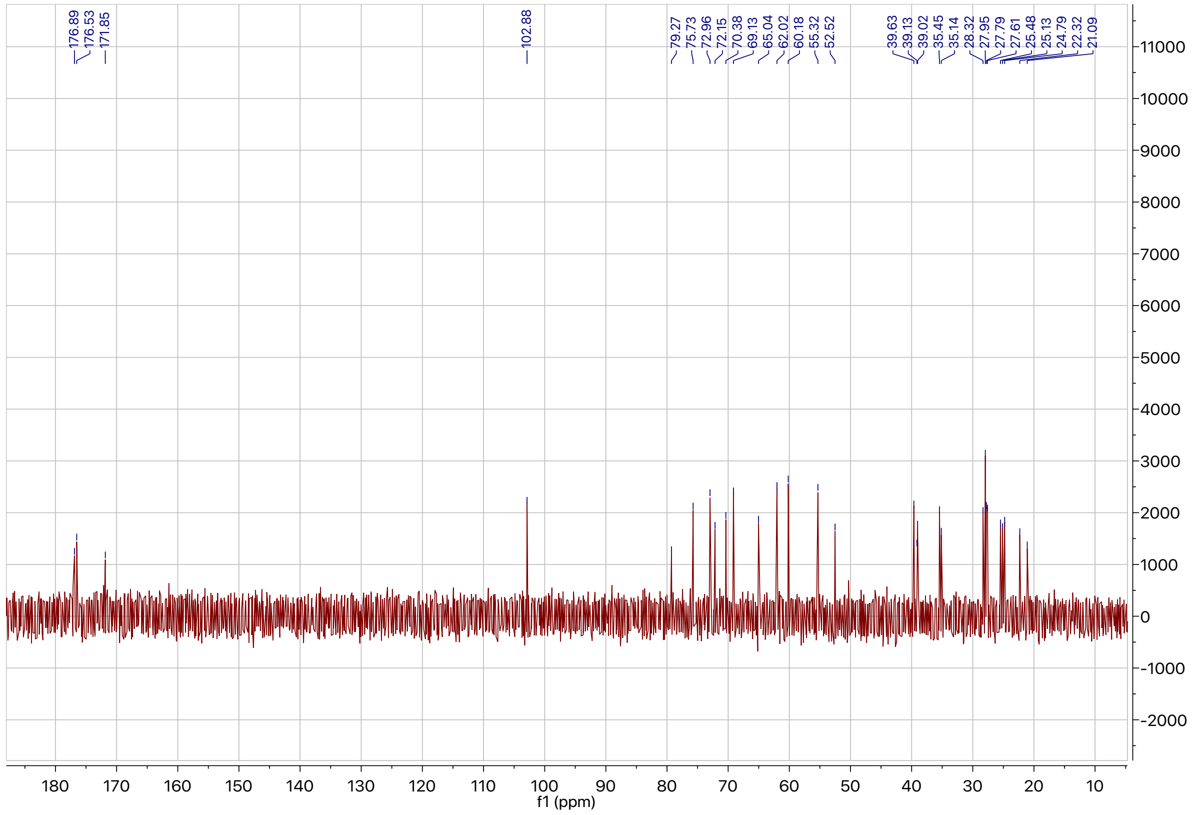

Supplementary Figure 11. ^{1}H (500 MHz, D_2O) and ^{13}C (126 MHz, D_2O) NMR spectra for **6**.

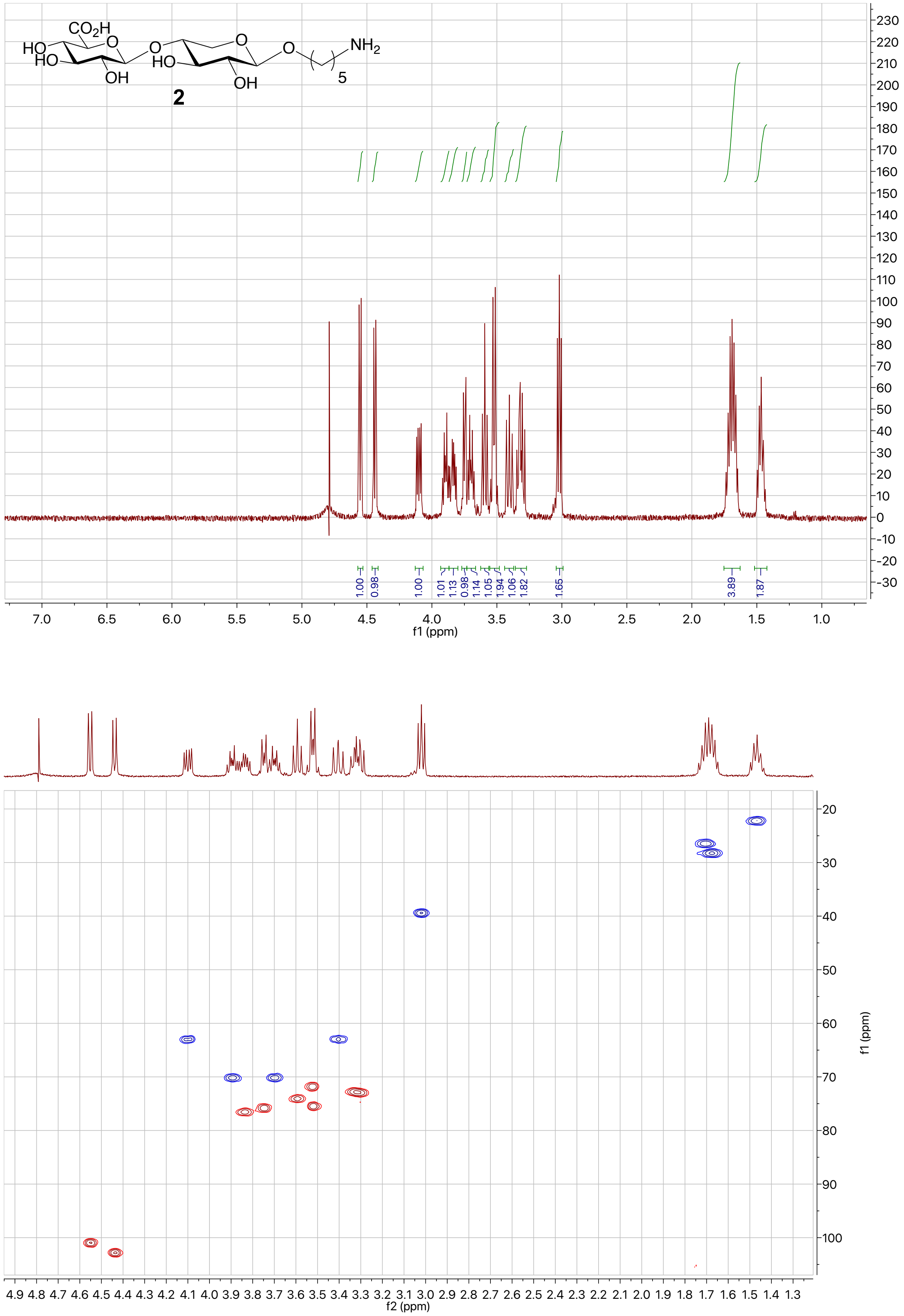

Supplementary Figure 12. ^{1}H (500 MHz, D_2O) and HSQC-DEPT (D_2O) NMR spectra for **2**.

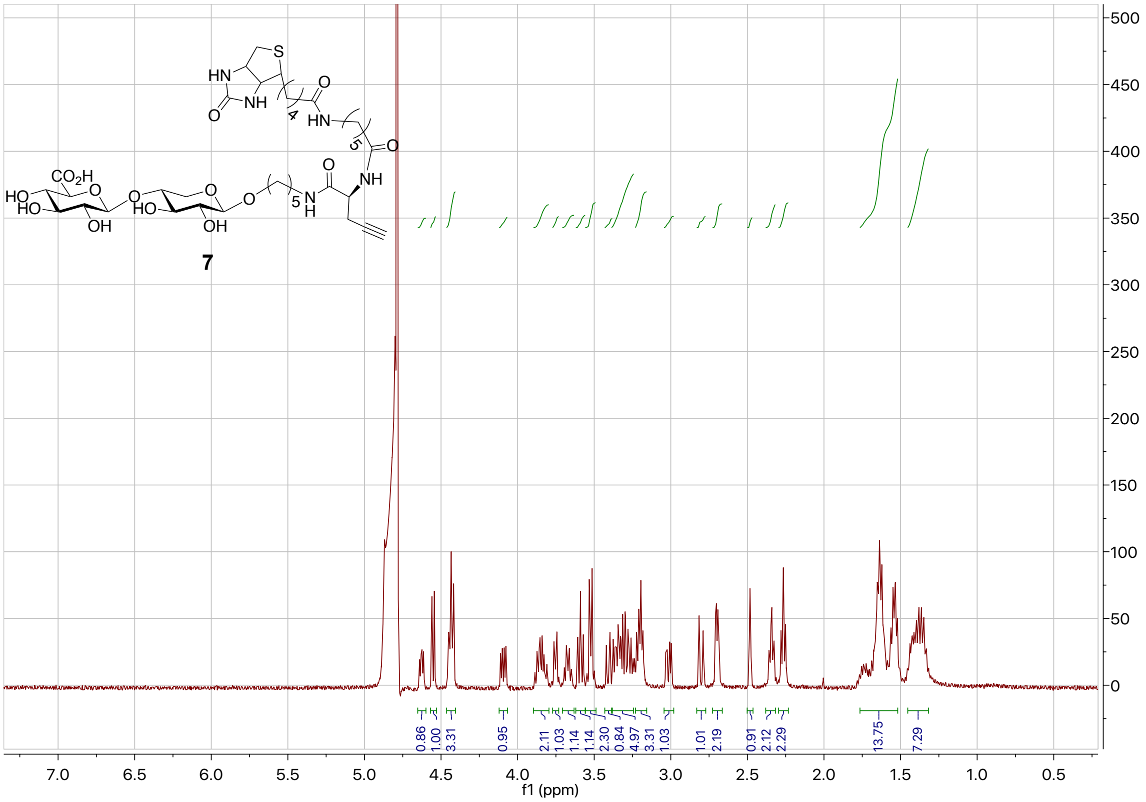

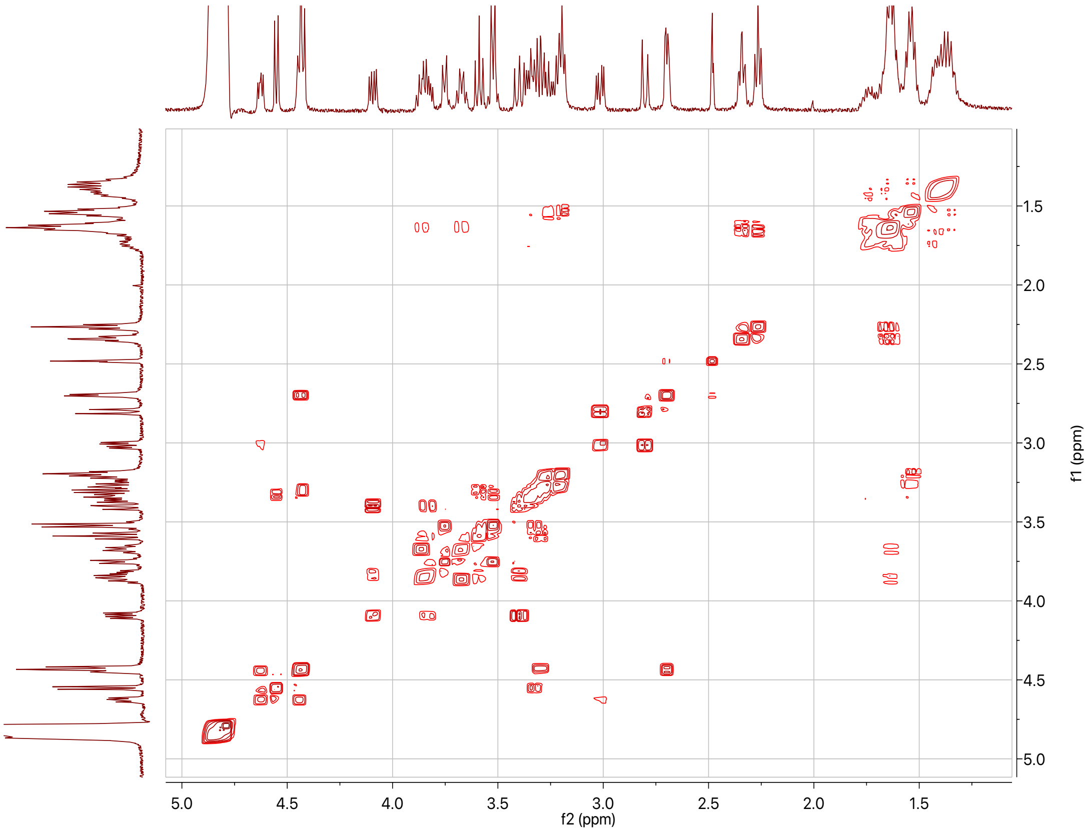

Supplementary Figure 13. ^{1}H (500 MHz, D_2O) and HSQC-DEPT (D_2O) NMR spectra for **7**.

Supplementary Figures: ESI-MS Data

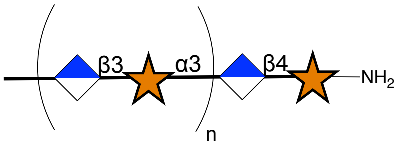

3a. n=2, **b**. n=3, **c**. n=4, **d**. n=5, **e**. n=6, **f**. n=7, **g**. n=8, **h**. n=9-11, **i**, n=12-14

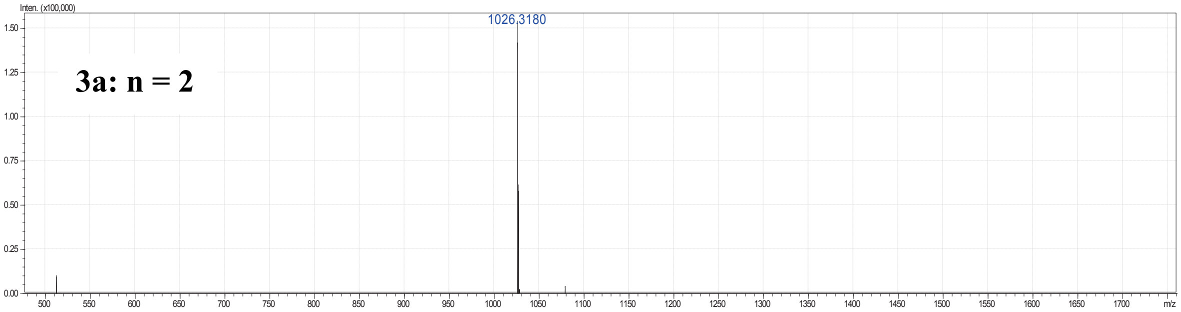

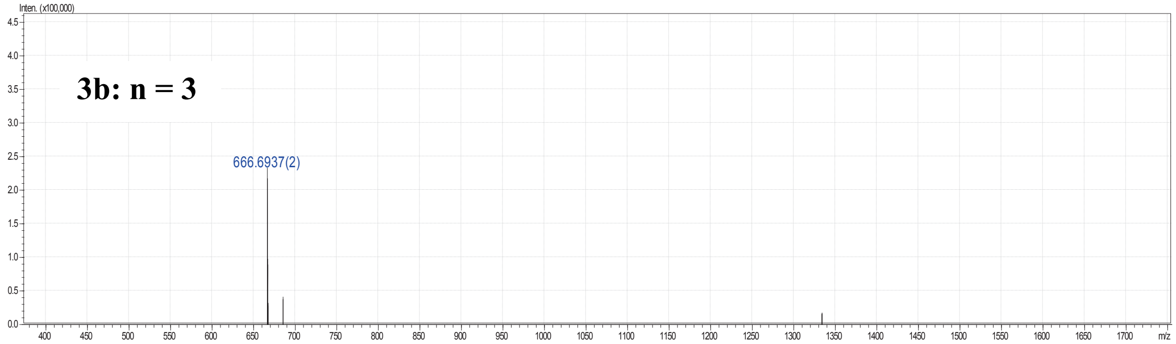

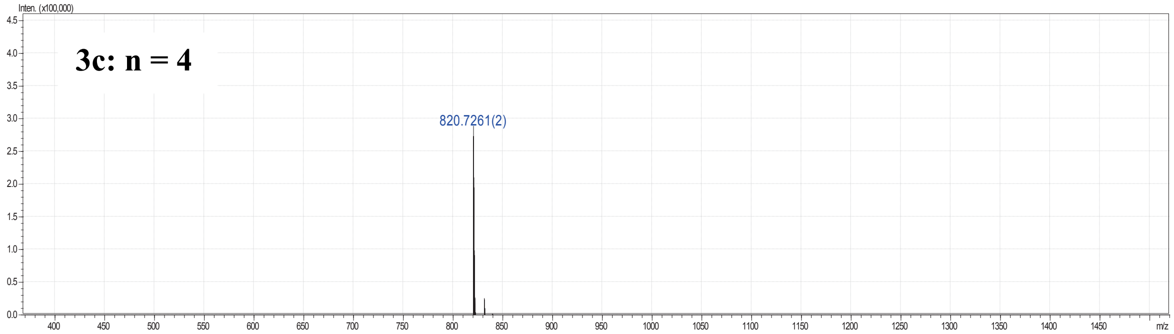

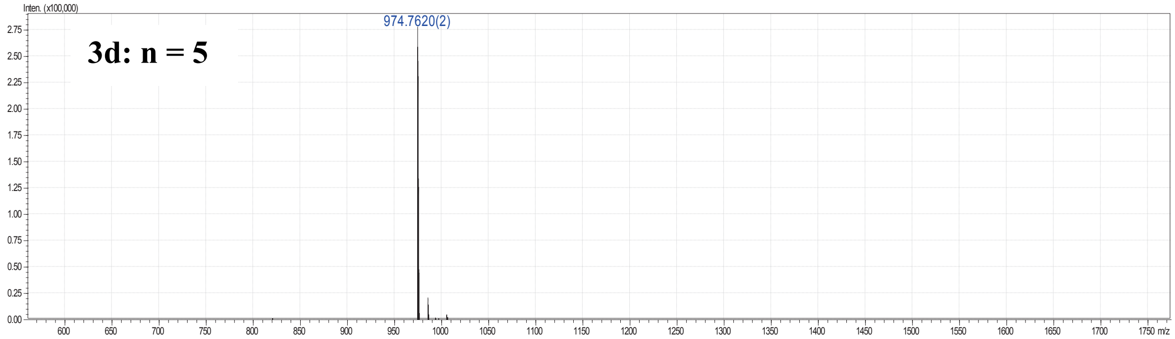

Supplementary Figure 14. ESI-MS data for **3a-d**.

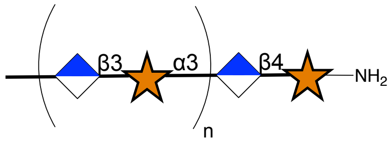

3a. n=2, **b**. n=3, **c**. n=4, **d**. n=5, **e**. n=6,
f. n=7, **g**. n=8, **h**. n=9-11, **i**, n=12-14

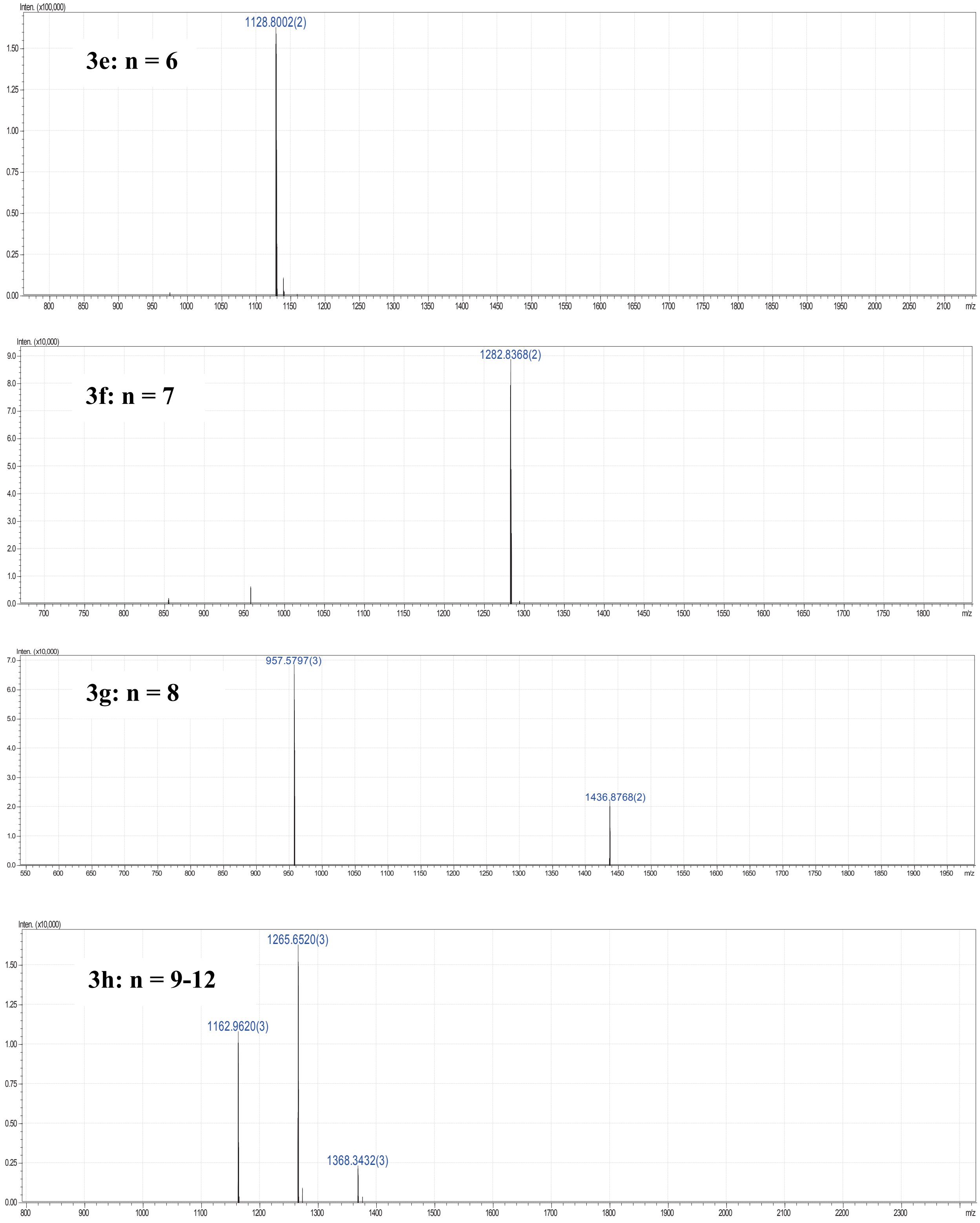

Supplementary Figure 15. ESI-MS data for **3e-h**.

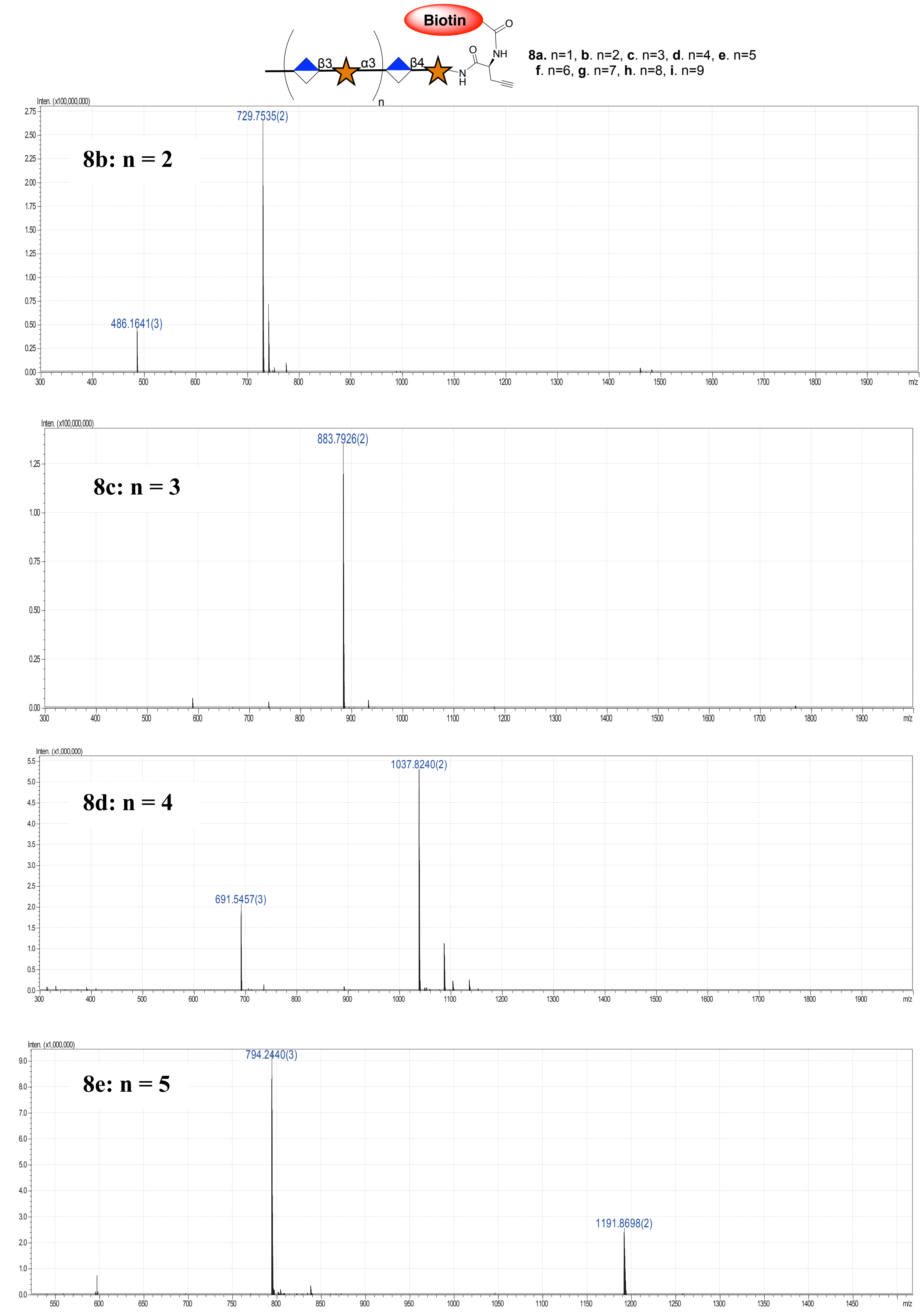

Supplementary Figure 16. ESI-MS data for **8b-e**.

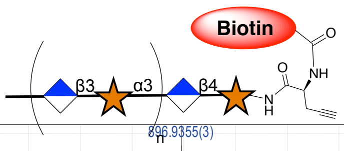

8a. n=1, **b**. n=2, **c**. n=3, **d**. n=4, **e**. n=5
f. n=6, **g**. n=7, **h**. n=8, **i**. n=9

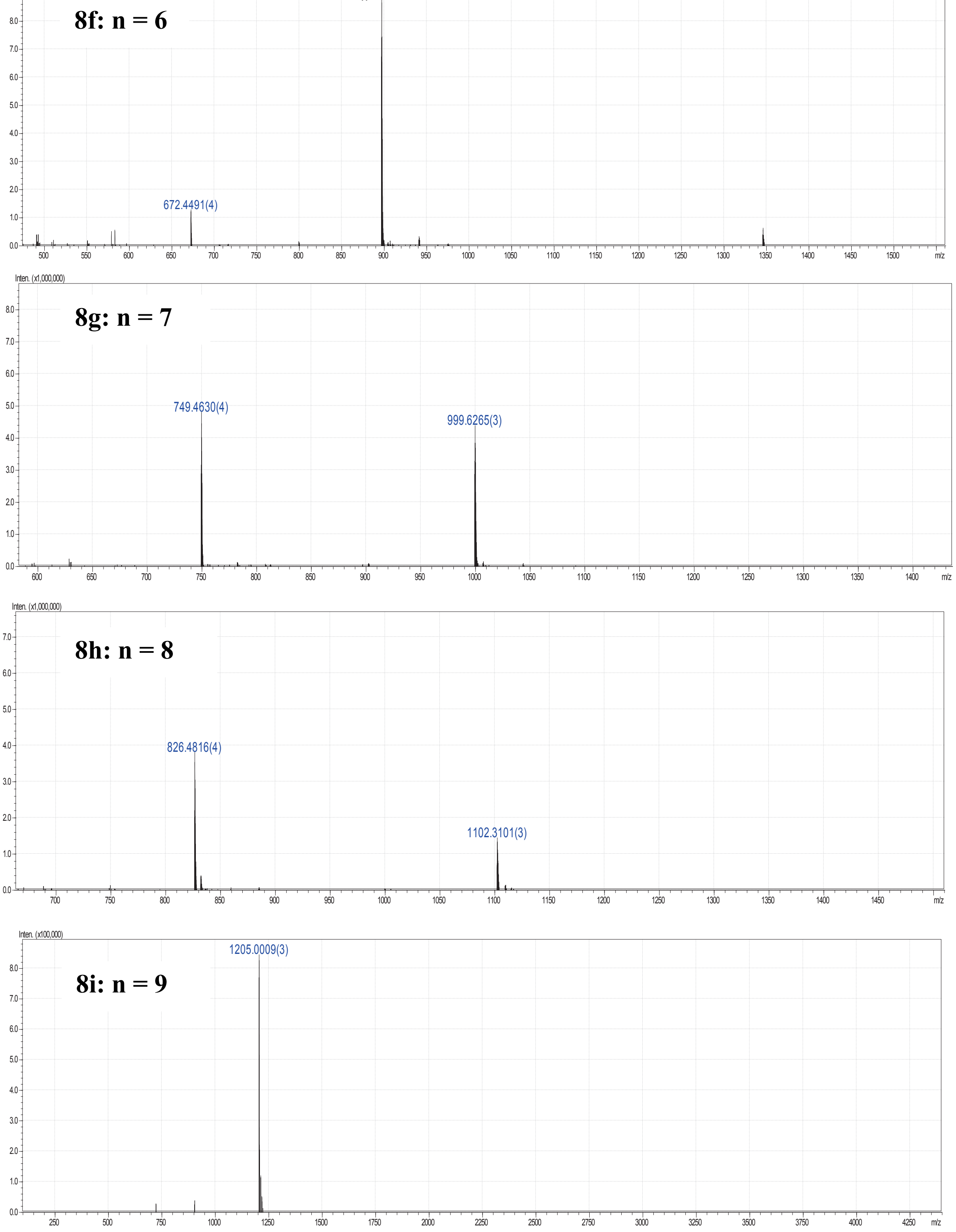

Supplementary Figure 17. ESI-MS data for **8f-i**.

Supplementary References

1 Capicciotti, C.J., *et al.* Cell-surface glyco-engineering by exogenous enzymatic transfer using a bifunctional CMP-Neu5Ac derivative. *J. Am. Chem. Soc.* **139**, 13342-13348 (2017).

2 Sun, T., *et al.* One-step selective exoenzymatic labeling (SEEL) strategy for the biotinylation and identification of glycoproteins of living cells. *J. Am. Chem. Soc.* **138**, 11575-11582 (2016).

3 Liu, L., Prudden, A.R., Bosman, G.P. & Boons, G.J. Improved isolation and characterization procedure of sialylglycopeptide from egg yolk powder. *Carbohydr. Res.* **452**, 122-128 (2017).
