# Supplementary material for: Cell surface glycan engineering reveals that matriglycan alone can recapitulate dystroglycan binding and function
Source: Nat Commun. 2022 Jun 24;13:3617. doi: 10.1038/s41467-022-31205-7 (PMC9232514; doi:10.1038/s41467-022-31205-7)
Supplement: Supplementary file 3 — Reporting Summary [file 41467_2022_31205_MOESM3_ESM.pdf]

nature research

Corresponding author(s): Boons, Geert-Jan
Wells, Lance

Last updated by author(s): Apr 28, 2022

Reporting Summary

Nature Research wishes to improve the reproducibility of the work that we publish. This form provides structure for consistency and transparency in reporting. For further information on Nature Research policies, see our Editorial Policies and the Editorial Policy Checklist.

Statistics

For all statistical analyses, confirm that the following items are present in the figure legend, table legend, main text, or Methods section.

n/a	Confirmed	
☐	☒	The exact sample size (*n*) for each experimental group/condition, given as a discrete number and unit of measurement
☐	☒	A statement on whether measurements were taken from distinct samples or whether the same sample was measured repeatedly
☐	☒	The statistical test(s) used AND whether they are one- or two-sided<br>*Only common tests should be described solely by name; describe more complex techniques in the Methods section.*
☒	☐	A description of all covariates tested
☒	☐	A description of any assumptions or corrections, such as tests of normality and adjustment for multiple comparisons
☐	☒	A full description of the statistical parameters including central tendency (e.g. means) or other basic estimates (e.g. regression coefficient) AND variation (e.g. standard deviation) or associated estimates of uncertainty (e.g. confidence intervals)
☐	☒	For null hypothesis testing, the test statistic (e.g. *F*, *t*, *r*) with confidence intervals, effect sizes, degrees of freedom and *P* value noted<br>*Give P values as exact values whenever suitable.*
☒	☐	For Bayesian analysis, information on the choice of priors and Markov chain Monte Carlo settings
☒	☐	For hierarchical and complex designs, identification of the appropriate level for tests and full reporting of outcomes
☒	☐	Estimates of effect sizes (e.g. Cohen's *d*, Pearson's *r*), indicating how they were calculated

Our web collection on statistics for biologists contains articles on many of the points above.

Software and code

Policy information about availability of computer code

Data collection	Microarray Data Collection: GenePix 4000B microarray scanner (Molecular Devices).<br>Fluorescent images were visualized using a fluorescence microscope (Nikon Eclipse, TE2000-S) and captured using a Qimaging (Retiga 1300i Fast) camera and Qcapture version 2.90.1 software.<br>Nexcelom K2 Cellometer.<br>Thermo Xcalibur Instrument Setup (v3.0)<br>Flow cytometry data collection was obtained on a Beckman Coulter HyperCyAn (CyAn ADP, Beckman Coulter, Hialeah, Florida) at the CTEGD Cytometry Center (University of Georgia)
Data analysis	Microarray Data Analysis: GenePix Pro 7 software (version 7.2.29.2, Molecular Devices).<br>ThermoFisher Scientific Proteome Discoverer v1.4<br>Premier Biosoft ProteoIQ (v2.7)<br>Fluorescence Images were unprocessed.<br>Raw data values were analyzed and plotted using GraphPad Prism 9.<br>FlowJo (v.8)

For manuscripts utilizing custom algorithms or software that are central to the research but not yet described in published literature, software must be made available to editors and reviewers. We strongly encourage code deposition in a community repository (e.g. GitHub). See the Nature Research guidelines for submitting code & software for further information.

Data

Policy information about availability of data

All manuscripts must include a data availability statement. This statement should provide the following information, where applicable:

- Accession codes, unique identifiers, or web links for publicly available datasets
- A list of figures that have associated raw data
- A description of any restrictions on data availability

The authors confirm that the data supporting the findings of this study are available within the article and/or its supplementary materials.

Data availability
The data that support the findings of this study are available from the corresponding authors, G.J.B. and L.W., upon reasonable request. The mass spectrometry proteomics data have been deposited to the ProteomeXchange Consortium (http://proteomecentral.proteomexchange.org) via the PRIDE partner repository with the dataset identifier PXD024251. Other data generated or analyzed during this study are included in this published article (and its Supplementary Information files).

Field-specific reporting

Please select the one below that is the best fit for your research. If you are not sure, read the appropriate sections before making your selection.

☒ Life sciences ☐ Behavioural & social sciences ☐ Ecological, evolutionary & environmental sciences

For a reference copy of the document with all sections, see nature.com/documents/nr-reporting-summary-flat.pdf

Life sciences study design

All studies must disclose on these points even when the disclosure is negative.

Sample size	All experiments were performed at technical triplicate or greater, and is appropriate for non-clinical, in vitro studies.
Data exclusions	Data were not excluded from analysis.
Replication	All experiments were performed at technical triplicate or greater, and successful.
Randomization	Randomization was not relevant to our study since this was non-clinical/in vitro work.
Blinding	Blinding was not relevant to our study since this was non-clinical/in vitro work.

Reporting for specific materials, systems and methods

We require information from authors about some types of materials, experimental systems and methods used in many studies. Here, indicate whether each material, system or method listed is relevant to your study. If you are not sure if a list item applies to your research, read the appropriate section before selecting a response.

Materials & experimental systems

n/a	Involved in the study	
☐	☒	Antibodies
☐	☒	Eukaryotic cell lines
☒	☐	Palaeontology and archaeology
☒	☐	Animals and other organisms
☒	☐	Human research participants
☒	☐	Clinical data
☒	☐	Dual use research of concern

Methods

n/a	Involved in the study	
☒	☐	ChIP-seq
☐	☒	Flow cytometry
☒	☐	MRI-based neuroimaging

Antibodies

Antibodies used

For microarray screening:
The mouse anti-glyco-alpha-dystroglycan antibody IIH6 (EMD Millipore) was used at final concentration of 5 µg/mL. IIH6 was detected by incubating the slide with 10 µg/mL goat anti-mouse Ig-Alexa Fluor 633 (Invitrogen A21046).
Biotinylated mouse-anti-His antibody (final concentration 10 µg/mL). GP-1 was detected by incubating the slide with 2 µg/mL of goat-anti-mouse-IgG (H+L) Alexa Fluor 633 antibody (Invitrogen A21050).

Flow Cytometry:

For analysis of IIH6 binding, matriglycan-engineered cells were incubated with the anti-glyco-alpha-dystroglycan antibody IIH6 (1/250) in 1% FBS/DPBS for 30 min at 4 °C. Cells were washed, then incubated with goat anti-mouse IgM conjugated with AlexaFluor-488 (1/100; Invitrogen A21046) for 30 min at 4 °C in the dark.

Immunoblotting and Laminin Overlay Assay:
Following SDS-PAGE, proteins were transferred to PVDF-FL (Millipore), blocked with Odyssey Blocking Buffer (Li-Cor), and probed with various antibodies as follows: The anti-α-DG core primary antibody (Goat 20 AP, 1:100 Dilution) was detected by secondary antibody donkey anti-goat IgG IR800CW (1:4000, Li-Cor). The anti-glyco α-DG7 primary antibody IIH6 [1:1000 Dilution (EMD Millipore)] was detected by secondary antibody goat anti-mouse IgM IR800CW (1:4000, Li-Cor). The anti-core β-DG mAb 7D11 (1:1000, Santa Cruz) was detected by secondary antibody donkey anti-mouse IgG IR680RD (1:10,000, Li-Cor).

Laminin overlay assays were performed as previously described, except recombinant His8-GFP-Lama1 was used. Briefly, following SDS-PAGE, proteins were transferred to PVDF-FL (Millipore), blocked for 1 hour with 5% Nonfat Dry Milk in Laminin Binding Buffer [LBB: 10 mM Triethanolamine (TEOA)-HCl pH 7.6, 140 mM NaCl, 1 mM CaCl2 and 1 mM MgCl2], and incubated with 10 μg/mL His8-GFP-Lama1 and 3% BSA in LBB, overnight at 4°C on an orbital shaker. The following day, membranes were washed in LBB and His8-GFP-Lama1 was detected using the anti-His.H8 antibody (1:1000, Millipore Sigma), followed by the secondary antibody donkey anti-mouse IgG IR680RD (1:2000, Li-Cor). All immunoblots were imaged using a Li-Cor Odyssey scanner.

LC-MS/MS proteomic analysis:
Clarified lysates were immunoprecipitated using protein G beads (Sigma-Aldrich) coated with unconjugated anti-biotin antibody (Jackson ImmunoResearch Laboratories 200-002-211).

Validation

Antibodies were validated by their respective suppliers and reported online.

Eukaryotic cell lines

Policy information about cell lines

Cell line source(s)

HAP1 Cells: Horizon Discovery. FreeStyle™ 293-F Cells (HEK293F) Cells: ThermoFisher

Authentication

None of the cell lines have been authenticated.

Mycoplasma contamination

Cell lines were not tested for mycoplasma contamination but no indication of contamination was observed.

Commonly misidentified lines
(See ICLAC register)

No commonly misidentified cell lines were used.

Flow Cytometry

Plots

Confirm that:

- [x] The axis labels state the marker and fluorochrome used (e.g. CD4-FITC).
- [x] The axis scales are clearly visible. Include numbers along axes only for bottom left plot of group (a 'group' is an analysis of identical markers).
- [x] All plots are contour plots with outliers or pseudocolor plots.
- [x] A numerical value for number of cells or percentage (with statistics) is provided.

Methodology

Sample preparation

For detection of the biotin handle using avidin, matriglycan-engineered cells were stained with avidin- AlexaFluor-488 (2.5 μg/mL; Invitrogen A21370) in 1% FBS/DPBS for 20 min at 4 °C in the dark. The cells were washed with DPBS without Ca/Mg, then detached using 150 μL of cell dissociation buffer for 2 min at 37 °C. The cells were suspended in 1% FBS/DPBS, centrifuged gently (500 rpm for 3 min), and resuspended in 500 μL of 1% FBS/DPBS and transferred to polystyrene tubes for flow cytometric analysis. Avidin-AlexaFluor-488 binding was determined by fluorescence intensity on the FL1(530/30 BP filter) emission channel. Data points were collected in duplicates and are representative of two separate experiments (n = 4).

For analysis of IIH6 binding, matriglycan-engineered cells were incubated with the anti-glyco-alpha-dystroglycan antibody IIH6 (1/250) in 1% FBS/DPBS for 30 min at 4 °C. Cells were washed, then incubated with goat anti-mouse IgM conjugated with AlexaFluor-488 (1/100) for 30 min at 4 °C in the dark. Cells were washed with DPBS without Ca/Mg and were detached, resuspended and analyzed as described above. Data points were collected in duplicates and are representative of two separate experiments (n = 4).

Instrument

Beckman Coulter HyperCyAn, CTEGD Cytometry Center, University of Georgia

Software

FlowJo software (Ashland, OR: Becton, Dickinson and Company)

Cell population abundance

Cell viability was determined by adding PI to cell suspensions 5 min prior to analysis. Avidin-AlexaFluor-488 binding was determined by fluorescence intensity on the FL1(530/30 BP filter) emission channel.

Gating strategy

The live population of cells was gated based on forward and side scatter emission, and exclusion of PI positive cells on the FL3

(613/20 BP filter) emission channel.

☒ Tick this box to confirm that a figure exemplifying the gating strategy is provided in the Supplementary Information.
